# Digital Outcomes of Upper Limb Ataxia Capture Meaningful Longitudinal Change and Treatment Response

**DOI:** 10.1002/mds.70012

**Published:** 2025-09-01

**Authors:** Dominik Hermle, Robin Schubert, Pascal Barallon, Winfried Ilg, Rebecca Schüle, Ralf Reilmann, Matthis Synofzik, Andreas Traschütz

**Affiliations:** ^1^ Division Translational Genomics of Neurodegenerative Diseases Hertie‐Institute for Clinical Brain Research and Center of Neurology, University of Tübingen Tübingen Germany; ^2^ George‐Huntington‐Institute Münster Germany; ^3^ Section Computational Sensomotorics, Hertie Institute for Clinical Brain Research Tübingen Germany; ^4^ Centre for Integrative Neuroscience (CIN) Tübingen Germany; ^5^ Division of Neurodegenerative Diseases and Movement Disorders, Department of Neurology Heidelberg University Hospital and Faculty of Medicine Heidelberg Germany; ^6^ Department of Neurodegeneration Hertie Institute for Clinical Brain Research, University of Tübingen Tübingen Germany; ^7^ Department of Clinical Radiology University of Münster Münster Germany; ^8^ German Center for Neurodegenerative Diseases (DZNE) Tübingen Germany

**Keywords:** ataxia; clinical outcome assessment; digital motor outcome; patient meaningfulness; sensitivity to change

## Abstract

**Background:**

Digital‐motor outcomes promise better responsiveness than clinician‐reported outcomes in ataxia trials. However, their patient meaningfulness and sensitivity to change remain to be demonstrated, particularly in the upper limb domain.

**Objectives:**

Validation of quantitative motor (Q‐Motor) assessment for upper limb ataxia against patient‐reported outcomes and regarding sensitivity to both longitudinal and treatment‐induced change, the latter in n‐of‐1 treatment settings.

**Methods:**

Single‐center longitudinal assessment of finger tapping, diadochokinesia, grip‐lift, spiral drawing, and target reaching in (1) 36 cross‐genotype ataxia patients and 20 controls, validating digital measures for correlations with patient‐reported outcome measure (PROM)‐ataxia, 2‐weeks test–retest reliability, and sensitivity to change within a trial‐relevant 1‐year follow‐up, anchored in Patient Global Impression of Change (PGI‐C); and (2) two patients with spinocerebellar ataxia type 27B (SCA27B) on versus off treatment with 4‐aminopyridine.

**Results:**

Twenty‐four digital measures correlated with the PROM‐ataxia upper‐limb composite (|ρ| = 0.4–0.7) and had excellent test–retest reliability (ICC = 0.91–0.99). Correlations to individual PROM‐ataxia items were specific for functional impairment the respective measure was hypothesized to capture. Speed of finger tapping and diadochokinesia, and smoothness of target reaching (spectral arc length of movement in three dimensions [SPARC_3D_]) captured 1‐year progression in ataxia patients (|*r*_prb_| = 0.38–0.51), and specifically in patients with worsening PGI‐C. Estimated sample sizes to detect longitudinal change were lower for digital than clinical outcomes (SPARC_3D_: n = 33, Scale for the Assessment and Rating of Ataxia (SARA): n = 79, nine‐hole peg‐test: n = 214). Speed of diadochokinesia, stability of grip‐lift, and variability of target reaching captured treatment responses to 4‐aminopyridine in SCA27B, exceeding minimal detectable and minimal important change.

**Conclusion:**

Digital upper limb measures capture patient‐meaningful 1‐year longitudinal and treatment‐induced change, and are therefore promising outcomes for upcoming ataxia trials. © 2025 The Author(s). *Movement Disorders* published by Wiley Periodicals LLC on behalf of International Parkinson and Movement Disorder Society.

With the prospect of molecular treatments for many genetic ataxias, sensitive outcome measures are urgently needed to conduct interventional trials. In the gait and balance domain, digital‐motor outcomes promise better sensitivity than clinician‐reported outcomes of ataxia, as recent longitudinal studies in spinocerebellar ataxia (SCA) type 2 and 3 have shown that digital gait measures could reduce trial sizes by at least 50% as compared to standard clinical assessment with the Scale for the Assessment and Rating of Ataxia (SARA).^1^, ^2^ In the upper limb domain of ataxia, digital‐motor outcomes have demonstrated promising construct validity in cross‐sectional studies using various tasks and assessments, for example, tapping tasks,^3^, ^4^ drawing tasks,^5^, ^6^, ^7^ tracking of (virtual) arm movements,^3^, ^8^, ^9^ or wearable and instrumented sensors.^10^, ^11^, ^12^, ^13^, ^14^ The respective digital upper limb measures were able to comprehensively capture the severity of upper limb ataxia in each clinician‐reported outcomes, performance outcomes, and activities of daily living.^3^, ^8^, ^15^ However, their key and trial‐relevant validation—that is, validation of their sensitivity to longitudinal change or change to drug treatment—is largely missing for digital measures of upper limb ataxia.^13^

Besides sensitivity to change, patient meaningfulness presents another challenge for digital‐motor outcomes of ataxia, including those for upper limb ataxia. Validation studies commonly infer that digital‐motor outcomes capture patient‐relevant impairment by cross‐correlating digital measures with clinical outcomes of functional impairment, for example, balance scales, activities of daily living, or performance outcomes in the affected motor domain.^3^, ^7^, ^8^, ^16^ However, although these correlations suggest patient meaningfulness, they do not sufficiently fulfill regulatory requirements for patient‐focused drug development.^17^ According to these requirements, patient meaningful outcomes must reflect specific health experiences of the patient (related to a concept of interest), and they must reflect clinically meaningful change.^17^ These requirements have not yet been met in validation studies of digital‐motor outcomes of ataxia in any domain.

The aim of this study was to validate novel digital‐motor measures for upper limb ataxia regarding their patient meaningfulness and sensitivity to change in two contexts of use: longitudinal progression‐related change and treatment‐induced change. The digital assessment comprises a quantitative motor (Q‐Motor) battery of finger tapping, diadochokinesia, grip‐lift, spiral drawing, and target reaching tasks and has recently been shown to capture multiple features of impaired upper limb movements in a large cross‐sectional and cross‐genotype ataxia cohort.^3^ Here, we conduct the longitudinal validation of these tasks and measures, including (1) correlations with patient‐reported outcome measure (PROM)‐ataxia and its individual upper limb items to demonstrate patient meaningfulness;^15^, ^18^ (2) its 2‐week test–retest reliability; and (3) its sensitivity to progression‐related change within a trial‐relevant 1‐year longitudinal follow‐up, anchored in Patient Global Impression of Change (PGI‐C). Moreover, sensitivity to treatment‐induced change was explored, as proof of concept, for (4) the setting of two n‐of‐1 treatments in spinocerebellar ataxia type 27B (SCA27B) assessed with versus without 4‐aminopyridine (4AP). Overall, this study shows that digital upper limb measures can capture both patient‐meaningful 1‐year longitudinal change and treatment‐induced change of upper limb ataxia, therefore, presenting a promising clinical outcome assessments for upcoming trials.

## Subjects and Methods

### Subjects

The study cohort comprised all 36 patients with an annual follow‐up visit at the ataxia clinic of the University Hospital of Tübingen after previous baseline Q‐Motor assessment.^3^ Patients had been included at baseline if they had degenerative cerebellar ataxia with or without sensory ataxia, and excluded if additional non‐cerebellar motor or cognitive impairment was severe enough to cause relevant additional non‐ataxia impairment (see Table S1 for a list of diagnoses). Age‐ and sex‐matched healthy controls had been recruited among patients' company, staff members, and medical students, and 20 healthy controls were re‐assessed as available at annual intervals. This study was approved by the institutional review board of the Medical Faculty of the University of Tübingen (824/2019BO2), and all subjects provided written informed consent.

### Digital‐Motor Assessment

All subjects were assessed with Q‐Motor (George‐Huntington‐Institute and QuantiMedis, Münster, Germany),^19^ comprising five motor tasks measured by force sensor and/or position field: (1) finger tapping; (2) diadochokinesia; and (3) grip‐lift (lifting and static holding of an object), performed and averaged over three successive trials with the dominant and non‐dominant hand, respectively; as well as (4) spiral drawing, and (5) visually directed target reaching between four targets on a board, performed as practice and test trial with the dominant hand, respectively.^3^ Upper limb ataxia was captured with 69 measures across six movement features based on previous cross‐sectional validation^3^: measures of slower speed and higher variability (in finger tapping, diadochokinesia, and target reaching, eg, based on tap intervals or spatiotemporal trajectories), lower positional stability (in grip‐lift, eg, based on unwanted drift or rotation), impaired smoothness (in spiral drawing and target reaching, eg, based on spectral arc length [SPARC]), and lower efficiency and endpoint precision (in target reaching, eg, as path length and dysmetria).

### Assessment of Clinician‐Reported Outcomes, Patient‐Reported Outcomes, and Other Performance Outcomes

For comparison with digital measures, several patient‐reported outcomes (PRO), clinician‐reported outcomes (ClinRO), and additional performance‐outcomes (PerfO) were assessed. Patient‐meaningful impairment was assessed cross‐sectionally by applying the PROM‐ataxia in a consecutive subset of 31 of 36 patients as soon as it became available in German. The PROM‐ataxia is a 70‐item questionnaire that asks for the frequency of specific ataxia‐related symptoms (eg, “My hands/arms shake and/or tremor when doing tasks,” with a 0–4 Likert scale from 0 = “never” to 4 = “always”) and the severity of specific functional impairment (eg, “I can brush my teeth without assistance,” with a 0–4 Likert scale from 0 = “without any difficulty” to 4 = “unable to do”) based on the previous 2‐week experience.^18^ It yields a total score (range: 0–280), domain scores, and an upper limb composite of 16 items (range: 0–64).^15^ Patient‐meaningful change was assessed longitudinally by asking for the PGI‐C relative to the time of baseline assessment, with stratification into three levels of “worsening,” “stable/no change,” and “improvement/better.”^20^ Other clinical outcome assessments of ataxia severity at baseline and annual follow‐up visits included the SARA (as ClinRO),^21^ the Activities of Daily Living part of the Friedreich Ataxia Rating Scale (FARS‐ADL; as PROM),^22^ and the nine‐hole peg‐test (9HPT; as PerfO).^3^

### Sensitivity to Treatment‐Induced Change

Sensitivity to change related to drug treatment was exemplarily explored, leveraging treatment effects of 4AP in SCA27B^23^, ^24^ as a showcase model. Specifically, Q‐Motor assessments were performed in two patients with SCA27B with and without 4AP (10 mg extended release, twice daily) assessed under a named patient treatment protocol.^25^ Both patients were first assessed under ongoing treatment, and then after a 4AP pause for 1 day (3 weeks later in patient 1; 3 days later in patient 2).

### Statistical Analysis

To determine convergent validity for patient meaningfulness, all 69 Q‐Motor measures were first correlated to the upper limb composite of the PROM‐ataxia,^15^ and all measures without significant correlation were discarded. The remaining measures were correlated to the nine single PROM‐ataxia items of the PROM‐ataxia upper limb composite, which exclusively reflect upper limb function—with the a priori hypothesis that patient meaningfulness would manifest in patterned correlations between task‐specific functional impairments and corresponding digital‐motor tasks and measures by which they are most likely captured (eg, legible writing by measures of spiral drawing; or tremor during task execution by measures of target reaching). The Bonferroni‐Holm method was used to adjust for multiple comparisons of overall 11 independent movement features across tasks. Spearman correlation was applied to account for the ordinal scale of PROM‐ataxia ratings and non‐parametric distributions of digital‐motor measures.

Test–retest reliability was validated in a subset of 31 subjects (14 ataxia patients, 17 healthy controls) that could be re‐assessed after an average 2‐week interval (median: 12 days, range: 7–30). Digital measures were kept if their test–retest analysis showed (1) an intraclass correlation coefficient (ICC) >0.9; (2) a smallest real difference <14% of a measure's range; and (3) a mean paired difference between test and re‐test (learning effect) <3% of a measure's range. These criteria were based on a data‐driven framework for digital outcome validation,^26^ applying even stricter cutoffs just sufficient to include at least one digital measure per task in the final set of most reliable measures. The minimal detectable change (MDC) for a 90% confidence level (MDC_90_) was calculated based on a measure's standard deviation (SD) in the retest cohort and a z‐score of 1.65 by

$${\mathrm{MDC}}_{90}=1.65\times \mathrm{SD}\times \left[\sqrt{2\;\left(1-\mathrm{ICC}\right)}\right].$$

Sensitivity to longitudinal change was analyzed by pairwise non‐parametric comparisons between baseline and 1‐year follow‐up assessments, using the Wilcoxon signed‐rank test with matched‐pairs rank biserial correlations (*r*_prb_) and bootstrapped 95% confidence intervals as measure of effect size.^1^, ^2^ Stepwise validation first required digital measures (and the ClinRO, PRO, and PerfO for comparison) to show significant deterioration only in ataxia patients, but no (biologically implausible) improvement in ataxia patients and no change in healthy controls (two‐sided tests). The absence of measures with opposite findings is reported. Measures with sensitivity to change in the full ataxia cohort were then further validated after stratification of patients by PGI‐C, with validation requiring significant longitudinal deterioration patients with subjective worsening of ataxia (n = 22), but no change in patients with subjective stability or improvement (one‐sided test) (n = 14). The average change in patients with worsening PGI‐C, and the 95% confidence interval of the change in patients with stable or improving PGI‐C were calculated as liberal and conservative estimates of the minimal important change (MIC) perceived by the patients (MIC based on average change [MIC_AC_] and MIC based on 95% confidence interval [MIC_95_]).^27^

As an illustrative method to compare sensitivity to longitudinal change between digital PerfOs versus other clinical outcome assessments, G*power 3.1 was used to estimate sample sizes required to detect longitudinal progression,^28^ modeling the detection of a difference from zero given the mean and SD of the 1‐year change across ataxia patients. All other tests were performed using MATLAB R2024a (The MathWorks, Natick, MA). For all analyses, the reciprocal transformation of the 9HPT^29^ and the log‐transformation of selected digital measures were used to account for non‐normal distributions revealed by Shapiro–Wilk testing and visual inspection of data.

## Results

Digital 1‐year follow‐up assessments (interval: 12 ± 1 months) were performed in 36 cross‐genotype ataxia patients (20 female; age: 53 ± 17 years, SARA: 11 ± 5; see Table S1 for patients and diagnosis) and 20 sex‐ and age‐matched controls (12 females, Fisher's exact test: *P* = 0.785; age: 49 ± 15 years, *t* test: *P* = 0.391). Patients with follow‐up assessments had less severe ataxia at baseline than patients lost to follow‐up (SARA: 11 ± 5 vs. 15 ± 7, *t* test, *P* = 0.017).

### Item‐Level Correlation to PROM‐Ataxia

**FIG. 1:**
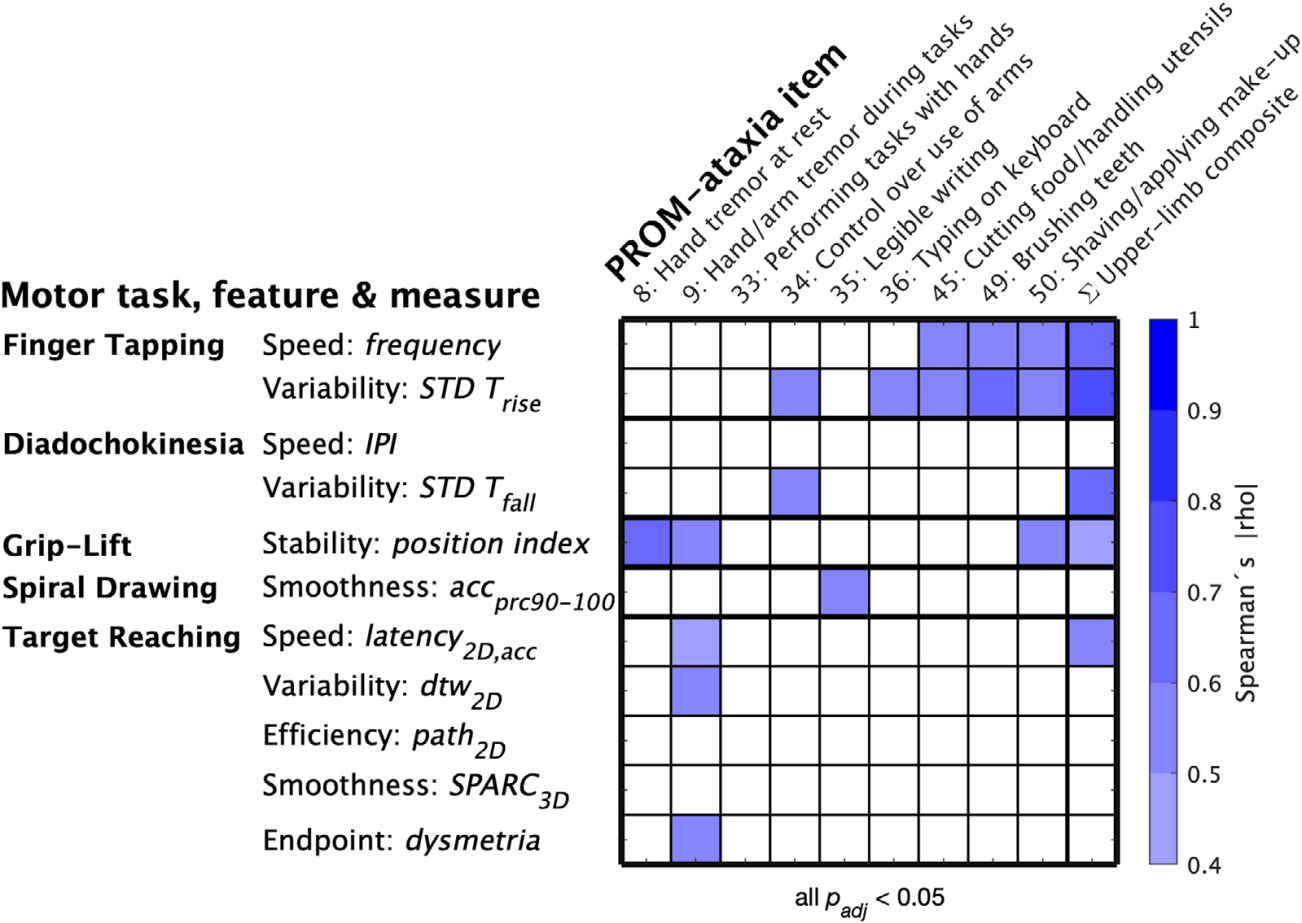
Item‐level correlation to PROM‐ataxia. Spearman correlation between representative digital measures of 11 movement features and individual PROM‐ataxia items that reflect specific impairment in upper limb function. Significance levels adjusted for multiple comparisons with the Bonferroni‐Holm method. PROM, patient‐reported outcome measure. [Color figure can be viewed at wileyonlinelibrary.com]

Forty‐two measures across all tasks correlated with the upper limb composite of the PROM‐ataxia (|ρ| = 0.36–0.70, all *P* < 0.05) (Table S2). They at least matched, but predominantly exceeded the correlation effect size of the 9HPT as standard PerfO for upper limb ataxia (non‐dominant hand: ρ = 0.38, *P* = 0.041; dominant hand: ρ = 0.27, *P* = 0.145). Item‐level correlations with PROM‐ataxia upper limb items were consistent with functional impairment hypothesized to be captured by the respective individual digital motor tasks and features (Fig. 1) (|ρ| = 0.50–0.65, all *P* < 0.05, adjusted for multiple comparisons) (see Table S2 for correlation coefficients between all items and measures). For example, illegible writing was exclusively correlated with irregular spiral drawing; functional impairment in distal fine motor tasks was associated with slow and irregular finger tapping; and patient‐reported tremor during task execution was specifically associated with positional instability during grip‐lift as well as slower speed, increased variability, and dysmetria in target reaching.

### Test–Retest Reliability

Validation criteria for test–retest reliability were met by 24 of 42 residual measures, including measures of speed in finger tapping, diadochokinesia and target reaching; positional stability during grip‐lift; smoothness in spiral drawing; and measures of variability, smoothness, and efficiency in target reaching (ICC = 0.91–0.99) (see Table S3 for all measures and criteria, including ICC and MDC_90_; see Fig. S1 for Bland–Altman plots).

### Sensitivity to Longitudinal Change

**FIG. 2:**
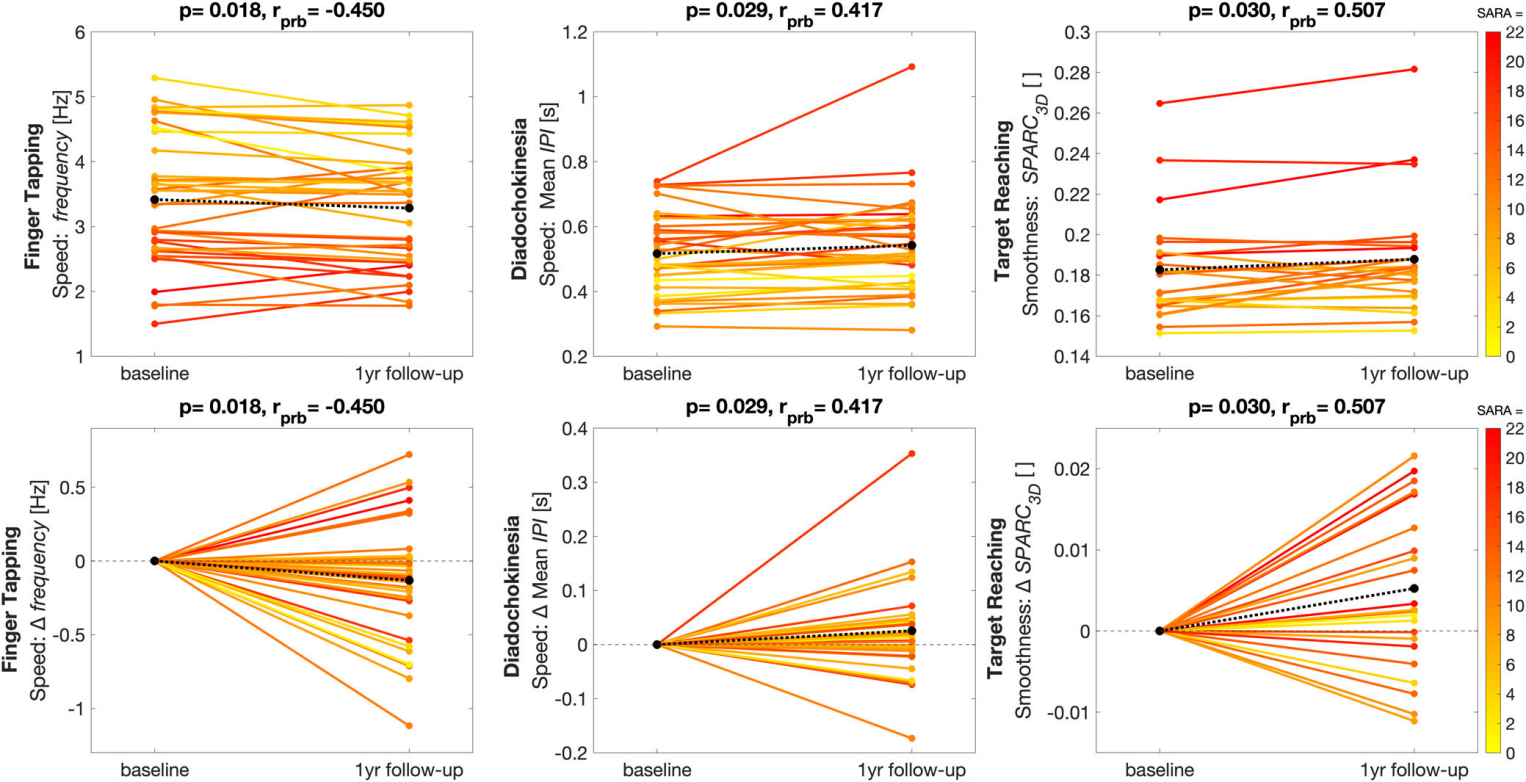
Within‐subject longitudinal change between baseline and 1‐year follow‐up, in absolute numbers (top panel) and change Δ (bottom panel). Baseline Scale for the Assessment and Rating of Ataxia scores of individual ataxia patients are color‐coded. Dashed line presents mean change across patients. Effect sizes *r*_prb_ determined by matched‐pairs rank biserial correlation. [Color figure can be viewed at wileyonlinelibrary.com]

Longitudinal progression of upper limb ataxia from baseline to 1‐year follow‐up was detected by 6 of 24 measures across three movement features and tasks: by slower speed of finger tapping (frequency: *r*_prb_ = −0.45; inter‐onset interval: *r*_prb_ = 0.38; tap duration: *r*_prb_ = 0.40) and diadochokinesia (frequency: *r*_prb_ = −0.38; inter‐peak interval: *r*_prb_ = 0.42), all in the non‐dominant hand; and decreased smoothness of target reaching (SPARC of movement in three dimensions [SPARC_3D_]: *r*_prb_ = 0.51, Wilcoxon signed‐rank test, all *P* < 0.05) (Table 1, Fig. 2). In turn, none of the measures detected improvement in ataxia patients, and only one of 24 measures changed in healthy controls (Table S4). When stratified by PGI‐C, measures of speed of finger tapping and diadochokinesia specifically decreased in patients with subjective worsening of ataxia at 1‐year follow‐up, but not in patients with subjective stability or improvement, therefore, reflecting patient‐meaningful change (Table 1, including the respective MIC_AC_ and MIC_95_). This was also observed for smoothness of spiral drawing (cumulative total of the highest decile of instantantenaous accelerations, *acc*_*prc90‐100*_: *r*_prb_ = 0.47 in progressors PGI‐C, *P* = 0.029), although no change was detected in the overall ataxia cohort (*P* = 0.222). In the cumulative distribution of changes, all these measures showed a systematic shift between subjectively progressive patients and healthy controls, whereas their changes in subjectively non‐progressive patients ranged from overlap with healthy controls (ie, capturing no change) to overlap with progressive patients (ie, capturing change not perceived by the patient) (Fig. S2).

**TABLE 1:** Sensitivity to longitudinal change

Task feature measure	Paired difference at 1‐year follow‐up						
	Healthy controls	Ataxia patients by PGI‐C					
		All		Progression		No progression	
	*r*_prb_	*r*_prb_ [95% CI]	m (SD)	*r*_prb_ [95% CI]	MIC_AC_	*r*_prb_	MIC_95_
Finger tapping ‐Speed							
Frequency [ndom]	n.s.	−0.45*[−0.74–0.06]	−0.13 (0.39)	−0.49* [−0.83 0.05]	−0.16	n.s.	−0.26
Mean IOI [ndom]	n.s.	0.38* [−0.03 0.68]	0.01 (0.05)	n.s.	–	n.s.	0.02
Mean IPI [ndom]	n.s.	n.s.	0.01 (0.05)	0.42* [−0.18 0.82]	0.01	n.s.	0.02
Mean TD [ndom]	n.s.	0.40* [0.00 0.70]	0.01 (0.03)	n.s.	–	n.s.	0.02
Diadochokinesia ‐ Speed							
Frequency [ndom]	n.s.	−0.38* [−0.68–0.01]	−0.09 (0.24)	−0.44* [−0.79 0.05]	−0.08	n.s.	−0.24
Mean IPI [ndom]	n.s.	0.42* [0.02 0.72]	0.03 (0.08)	0.42* [−0.09 0.79]	0.03	n.s.	0.05
Spiral drawing ‐ Smoothness							
*acc*_*prc‐90‐100*_	n.s.	n.s.	0.03 (0.27)	0.47* [−0.05 0.83]	0.08	n.s.	0.09
Target reaching ‐ Smoothness							
SPARC_3D_	n.s.	0.51* [0.03 0.81]	0.01 (0.02)	n.s.	–	n.s.	0.02
Clinical outcomes							
SARA	–	n.s.	0.75 (2.28)	0.54* [0.01 0.86]	0.91	n.s.	2.32
FARS ADL	–	0.49* [0.04 0.79]	0.96 (2.51)	0.48* [−0.12 0.85]	1.05	n.s.	2.14
Reciprocal 9HPT [dom]	0.65* [0.11 0.92]	n.s.	−0.001 (0.007)	n.s.	–	n.s.	0.002
Reciprocal 9HPT [ndom]	–	n.s.	0.001 (0.005)	n.s.	–	n.s.	0.004

Abbreviations: 9HPT, 9‐hole peg‐test; 95% CI, bootstrapped 95% confidence interval; acc, acceleration; dom, dominant hand; ndom, non‐dominant hand; FARS‐ADL, Friedreich Ataxia Rating Scale Activities of Daily Living; IOI, inter‐onset interval; IPI, inter‐peak interval; m, mean; MIC_AC_, minimal important change, based on average change; MIC_95_, minimal important change, based on 95% confidence interval; n.s., not significant; PGI‐C, Patient Global Impression of Change; SARA, Scale for the Assessment and Rating of Ataxia; SD, standard deviation; SPARC, spectral arc length; TD, tap duration.

* *p* < 0.05 in Wilcoxon signed rank test.

**FIG. 3:**
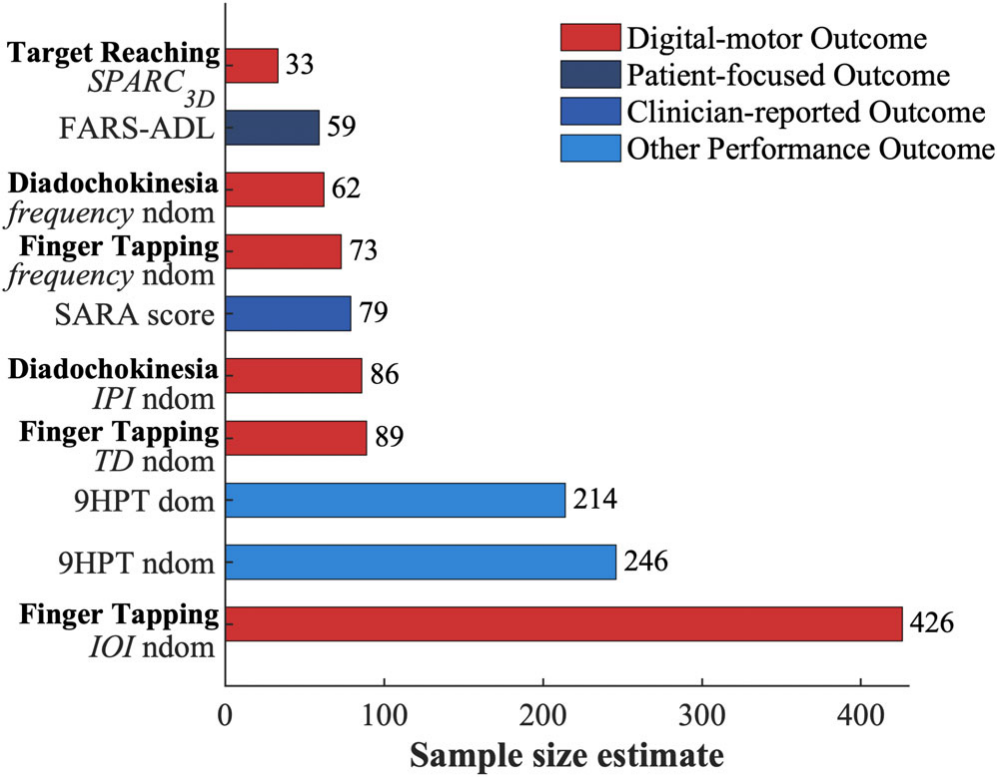
Sample size estimations for comparison of sensitivity to longitudinal change between digital measures and standard ataxia outcomes. Sample sizes required for significant detection of 1‐year longitudinal change based on mean and standard deviation of change across the full ataxia cohort. 9HPT, Nine‐Hole Peg‐Test; FARS‐ADL, Activities of Daily Living of Friedreich Ataxia Rating Scale; IOI, inter‐onset interval; IPI, inter‐peak interval; ndom, non‐dominant hand; SARA, Scale for the Assessment and Rating of Ataxia; SPARC, spectral arc length. [Color figure can be viewed at wileyonlinelibrary.com]

Among other clinical outcome assessments, longitudinal change at 1‐year follow‐up was not detected by the 9HPT as standard PerfO for upper limb ataxia (dominant hand: *P* = 0.118, non‐dominant hand: *P* = 0.390). Global progression of ataxia was captured by the FARS‐ADL in the full ataxia cohort (*r*_prb_ = 0.40, *P* = 0.028), and by the SARA specifically in patients with worsening PGI‐C (*r*_prb_ = 0.54, *P* = 0.016) (Table 1). A comparison of sensitivity to change of digital PerfOs versus other clinical outcome assessments by means of sample size estimations showed that smoothness of target reaching (SPARC_3D_) had the highest sensitivity to longitudinal change (Fig. 3). The corresponding sample size of 33 patients was approximately half the size required by the FARS‐ADL (n = 59) or the SARA (n = 79), which capture all motor domains of ataxia, not only upper limb ataxia. Sensitivity to longitudinal change of speed in diadochokinesia (frequency: n = 62) and finger tapping (frequency: n = 73) were comparable to the sensitivity of FARS‐ADL and SARA. All digital measures had markedly better sensitivity than the 9HPT (dominant hand: n = 214, non‐dominant hand: n = 246).

### Sensitivity to Treatment‐Induced Change

**FIG. 4:**
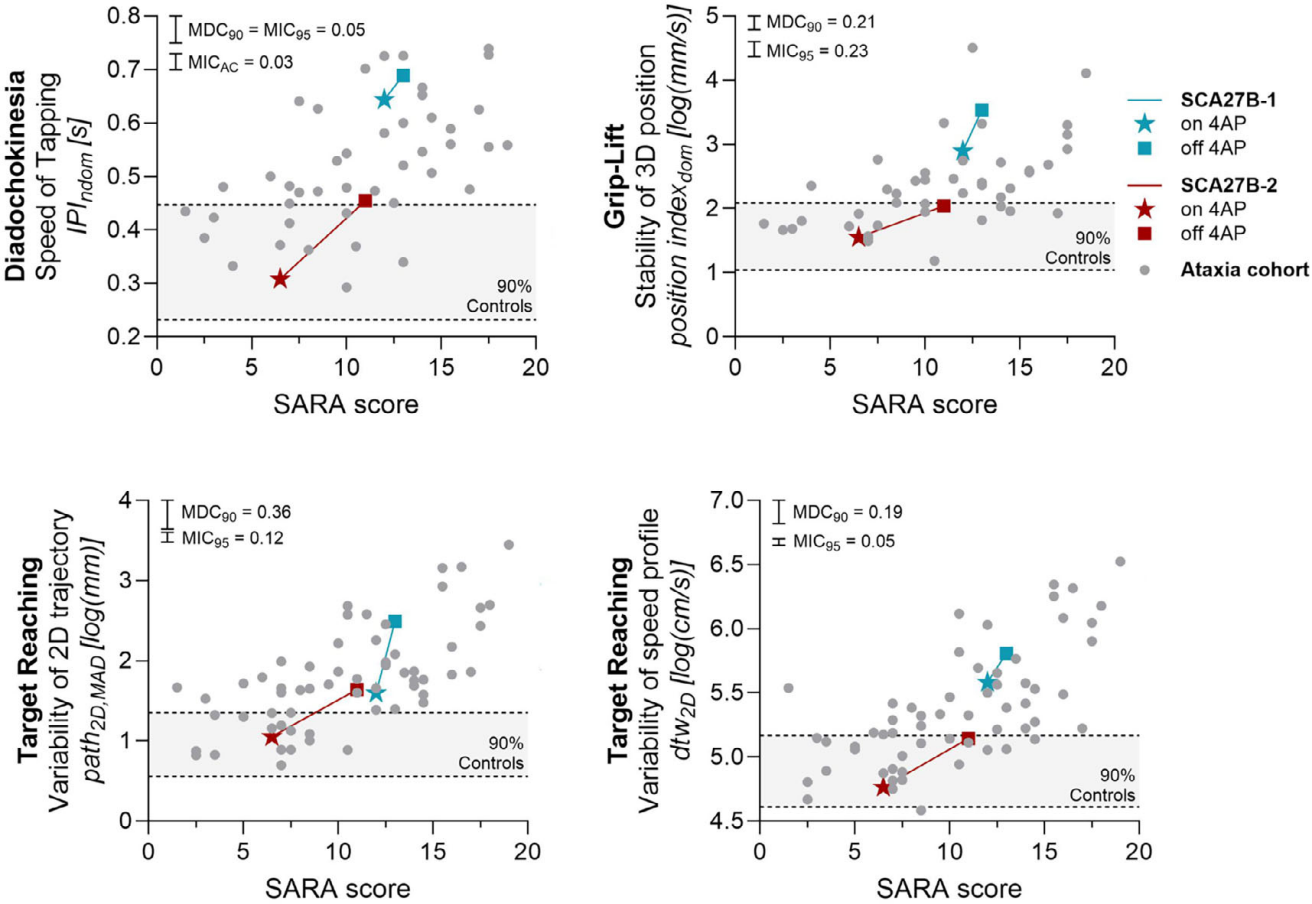
Sensitivity to drug treatment. Within‐subject change of digital measures in two SCA27B patients on versus off treatment with 4‐aminopyridine (4AP), plotted against the corresponding change of the SARA. Scattered dots present the distribution of digital measures relative to ataxia severity in the longitudinal validation cohort. Shaded areas display the corresponding 90th percentile in the control cohort. Bars indicate the size of the threshold for a minimal detectable change (MDC) and a minimal important change (MIC) based on the validation of test–retest reliability and sensitivity to longitudinal change, respectively. DTW, dynamic time warp of time‐speed series; IPI, inter‐peak interval; MAD, median absolute deviation; ndom, non‐dominant hand; SARA, Scale for the Asessment and Rating of Ataxia; SCA27B, spinocerebellar ataxia type 27B. [Color figure can be viewed at wileyonlinelibrary.com]

Withdrawal of 4AP in two patients with SCA27B was associated with slower speed of diadochokinesia (inter‐peak interval between taps), impaired stability of grip‐lift (position index), and increased variability of the spatial trajectory (median absolute deviation of path length in the two‐dimensional projection, path_2D,MAD_) and the time‐speed profile (dyamic time warp for profile of movement in two or three dimensions, dtw_2D/3D_) of target reaching, indicating sensitivity to drug treatment response (Fig. 4; see Fig. S3 for lack of sensitivity across other features). These measures worsened regardless of a small or a large deterioration in total SARA (1 and 4.5 points, respectively) or its composite of upper limb items (0.5 and 2 points) or a visible change in performance (see Fig. S4 for qualitative changes in target reaching) and exceeded the MDC_90_ threshold in both cases (error bars in Fig. 4). Their change also exceeded the MIC_95_ (calculated for sensitivity to longitudinal change) and was consistent with both patients' global impression of deterioration after 4AP withdrawal, therefore, reflecting patient‐meaningful change across several tasks and measures.

## Discussion

This study provides first validation of a digital‐motor assessment of upper limb ataxia for (1) patient meaningfulness, by item‐level correlation to specific upper limb impairment in the PROM‐ataxia, and by stratification of longitudinal change by PGI‐C; and (2) sensitivity to change in two contexts of use, that is, longitudinal progression‐related change within a trial‐relevant 1‐year interval, and responsiveness to drug treatment.

### Digital Measures Reflect Specific Patient Experience of Upper Limb Impairment

All clinical outcome assessments, including digital‐motor outcomes, should capture symptoms or impairment that are meaningful to patients. Accordingly, regulatory frameworks require patient‐meaningful outcomes to reflect specific health experiences of the patient related to a corresponding patient‐relevant concept of interest, for example, daily living functioning.^17^ To relate digital outcomes to such specific patient experiences, the present study correlated digital upper limb measures to individual upper limb items of the PROM‐ataxia, a patient‐reported outcome questionnaire on ataxia‐related functional impairment in daily living.^18^ Consistent with our a priori hypothesis, each motor task showed specific and plausible associations with patient‐reported impairment in daily living: speed and/or variability of finger tapping were correlated with items asking for distal fine motor skills (typing, cutting food/handling utensils, brushing teeth, and shaving/make‐up), while smoothness of spiral drawing was specifically associated with impaired writing. Further, grip‐lift stability was associated with patient‐reported tremor and impairment in a fine motor task that also requires stability of the arm (shaving/applying make‐up), whereas measures of target reaching correlated only with patient‐reported kinetic tremor. Patient‐meaningfulness of the diadochokinesia task remains unclear, for which measures of variability showed the highest correlations to the PROM‐ataxia upper limb composite and were specifically associated with patient‐reported loss of “control over the use of the arms.” We speculate that digital measures of “*dys*diadochokinesia”—a key feature of cerebellar pathophysiology—may reflect the patient's general (ie, cross‐task) experience of “loss of control” of upper limb coordination as a global genuine impairment because of cerebellar ataxia.

### Upper Limb Measures Capture Patient‐Meaningful 1‐Year Change

Sensitivity to longitudinal change—ideally exceeding that of standard ClinROs, PROs, and PerfOs—remains the key challenge in the development of digital‐motor outcomes for ataxia, so far accomplished only for digital gait measures.^1^, ^2^ This study demonstrates that the longitudinal progression of ataxia over a trial‐relevant 1‐year period can also be effectively captured by digital‐motor assessments in the upper limb domain, with sensitivity to change exceeding standard ataxia outcomes. Our sample size estimates, intended for comparative illustration to these other clinical outcome assessments rather than interpretation of absolute values, suggest that measures of speed in finger tapping and diadochokinesia, as well as smoothness in target reaching, are more sensitive to change than the SARA, which is the most commonly used ClinRO.^30^, ^31^, ^32^, ^33^ Given the limited sensitivity of the SARA's upper limb items and related regulatory concerns,^34^, ^35^ or their complete omission in the revised f‐SARA,^36^ these digital upper limb measures may serve as particularly promising digital‐motor outcomes to complement the SARA, especially in upcoming trials that aim to comprehensively capture ataxia, including upper limb impairment. Compared to the FARS‐ADL, a patient‐focused outcome across multiple motor and functional domains of ataxia,^22^, ^37^ digital speed measures achieved similar sensitivity based on the upper limb domain alone. For a targeted assessment of upper limb ataxia, the smoothness measure of target reaching could reduce trial sizes by even more than 80% compared to the 9HPT and eventually replace this commonly used upper limb performance outcome.^30^, ^31^, ^38^

For implementation as endpoint in clinical trials, digital‐motor outcomes must not only reflect patient‐meaningful impairment, but also capture patient‐meaningful change over time. By demonstrating sensitivity to change specifically in patients with subjective worsening (but not stability) in the PGI‐C as an external anchor, the present study demonstrated that a digital ataxia outcome could capture patient‐meaningful change within a trial‐relevant 1‐year interval. Moreover, this anchor‐based stratification allowed establishing a MIC threshold for all digital measures, which is key for the definition of an endpoint and its interpretation in a clinical trial. Such stratification of longitudinal change by PGI‐C has previously been used to identify patient‐meaningful changes of SARA and FARS‐ADL, including estimates for minimal important change.^37^, ^39^ With the first application to a digital ataxia outcome, our study now suggests that this approach could serve as a blueprint for other digital‐motor domains, where sensitivity to patient‐meaningful change remains to be validated.

### Upper Limb Measures Capture Treatment Response

This study next aimed to provide proof of concept for the sensitivity of digital outcomes to drug‐induced change of upper limb ataxia, particularly in n‐of‐1 treatment settings. To demonstrate such treatment responsivity, we used the efficacy of 4AP in SCA2B, which has now been increasingly substantiated.^23^, ^24^, ^25^ Indeed, digital measures of speed of diadochokinesia, stability of grip‐lift, and variability of target reaching worsened in two SCA27B patients after withdrawal of 4AP. Therefore, speed of diadochokinesia, that is, the digital assessment of a key examination of cerebellar dysfunction, captured both longitudinal change and drug‐induced change of upper limb ataxia. The selective sensitivity of grip‐lift stability and target reaching variability for treatment‐induced changes might have been because of a particular disease biology of SCA27B and/or a particular pharmacological effect of 4AP. For example, digital measures of postural tremor (grip‐lift: position index) or trial‐to‐trial variability and learning (target reaching: path_2D,MAD_, dtw_3D/2D_) may better reflect drug‐dependent compensatory mechanisms than the underlying progressive cerebellar degeneration. Therefore, additional studies covering other ataxia genotypes and treatments are needed to determine the appropriate context of use for these digital measures.

The 4AP‐related change in digital measures not only reflected the subjective worsening experienced by both patients, but also exceeded the respective thresholds for the MDC and the MIC. Although the unblinded open label treatment setting may have biased patient‐reported change, MIC_95_ and MIC_AC_ were independently derived from our longitudinal validation and, therefore, corroborate that the digital measures captured patient‐meaningful change. Notably, the digital measures also captured the treatment response despite generally mild upper limb involvement in SCA27B,^23^ and even in patient 2 whose degree of motor impairment was overlapping with the range of healthy controls (Fig. 4). This suggests that the digital upper limb outcomes identified here could not only be a useful trial outcome in advanced ataxia, when loss of ambulation prevents digital gait assessment, but also in the trial‐relevant mild ataxia stage when patients are still ambulatory.^3^

### Study Limitations

This study applied a stringent statistical analysis framework for measure selection and a conservative choice of cutoffs for a strict validation of digital measures. However, our findings are limited by the generic validation across different genetic ataxias. Their variable disease severity and progression limit the interpretation of absolute change values in our digital measures. Yet, this validation approach also enhanced the generalizability of our findings, and also included ultra‐rare ataxias, for which independent validation would in fact not be possible.^40^ Moreover, using a cohort that included different ataxia genotypes — and thus a broader range of progression rates — was probably key to show that digital measures can indeed capture longitudinal changes that are meaningful to patients. In a more homogeneous cohort with the same ataxia genotype and more similar progression, stratification by patient global impression of change might have been underpowered to detect patient‐meaningful differences in progression, at least in an early validation study. Additional studies in genetically stratified cohorts complementing our cross‐genotype study are required (and ongoing) in common trial‐relevant ataxias. These studies may reveal qualitatively different digital measures (eg, because of non‐ataxia involvement like bradykinesia or hyperkinetic movement disorders), and/or quantitatively different sensitivity to change per ataxia genotype. However, we expect our digital measures to be sensitive in trial‐relevant SCAs,^32^ Friedreich ataxia,^33^ or RFC1‐ataxia,^41^ because progression in these common genetic ataxia exceeds the average progression of 0.9 SARA points/year in our cohort with worsening PGI‐C. Our proof of concept of sensitivity of the digital upper limb outcome measures to drug treatment used withdrawal of 4AP as a previously applied paradigm in clinical trials,^42^ but it is—as proof of concept—limited by the small number of SCA27B patients and the unblinded paradigm. Further validation of the treatment‐responsivity of the digital motor outcomes identified here by larger, blinded, placebo‐controlled treatment trials is warranted. Nevertheless, the consistent treatment effect across several digital motor tasks and measures is unlikely to be caused by random variability or placebo effects. Effects of 4AP were only analyzed for digital measures that previously passed validation for high test–retest reliability, that is, measures with little variability to induce random effects. Drug‐related changes in all these measures either fell below the MDC or consistently indicated 4AP‐related improvement, but none of these measures indicated worsening that would be expected to occur by random variability. Other key arguments against a placebo effect are 4AP‐related changes in specific target reaching measures such as movement smoothness as well as between‐trial variability of time‐speed profiles (*dtw*). Although patient behavior or motivation may affect the overall speed or accuracy, the spatio‐temporal alignment of movement within and across multiple trial repetitions that underlies these measures is virtually impossible to manipulate by a naïve test subject.

## Conclusion

This study demonstrates that digital measures of upper limb ataxia allow capturing patient‐meaningful 1‐year longitudinal change across a wider set of degenerative ataxias, as well as responses to drug treatment. This indicates their promise as progression and treatment outcomes, respectively, in future natural history and treatment trials for a larger number of ataxias. Importantly, the sensitivity of digital measures to longitudinal change exceeded other clinical outcome assessments, including the SARA as standard ClinRO of ataxia severity, and the 9HPT as standard PerfO in the upper limb domain. Digital upper limb measures identified in this study could be rapidly implemented in upcoming ataxia trials given Q‐Motor's experience in several Huntington's disease trials^43^, ^44^, ^45^ and its availability at more than 150 sites worldwide, but also further validated using wearable sensors for the remote assessment of upper limb movements.

## Author Roles

(1) Article project: A. Conception, B. Organization, C. Execution. (2) Patient assessment: A. Execution. (3) Manuscript: A. Writing of the First Draft; B. Review and Critique.

D.H.: 1A, 1C, 2A, 3A, 3B

Ro.S.: 1A, 1B, 1C, 3B

P.B.: 1A, 1B, 1C, 3B

W.I: 1A, 3B

Re.S.: 1B, 3B

R.R.: 1A, 1B, 1C, 3B

M.S.: 1A, 1B, 1C, 3B

A.T.: 1A, 1B, 1C, 2A, 3A, 3B

## Supporting information


**Table S1.** Detailed patient characteristics.
**Table S2.** Correlations with PROM‐ataxia.
**Table S3.** Test‐retest reliability across digital motor tasks and measures.
**Table S4.** Sensitivity to longitudinal change across all digital measures validated for significant correlations with PROM‐ataxia and for test‐retest reliability.
**Fig. S1.** Bland‐Altmann plots of test‐retest assessment.
**Fig. S2.** Distribution of longitudinal change.
**Fig. S3.** 4AP‐related change in SCA27B across motor tasks and digital measures.
**Fig. S4.** 4AP‐related change on spatial trajectories of target reaching.

## Data Availability

Group level raw data will be shared on reasonable request by qualified investigators. No permission is available for sharing single subject‐level data.
