# Supplementary material for: Digital Outcomes of Upper Limb Ataxia Capture Meaningful Longitudinal Change and Treatment Response
Source: Mov Disord. 2025 Sep 1;40(11):2486–96. doi: 10.1002/mds.70012 (PMC12661631; doi:10.1002/mds.70012)
Supplement: Supplementary file 1 — Table S1. Detailed patient characteristics. Table S2. Correlations with PROM‐ataxia. Table S3. Test‐retest reliability across digital motor tasks and measures. Table S4. Sensitivity to longitudinal change across all digital measures validated for significant correlations with PROM‐ataxia and for test‐retest reliability. Fig. S1. Bland‐Altmann plots of test‐retest assessment. Fig. S2. Distribution of longitudinal change. Fig. S3. 4AP‐related change in SCA27B across motor tasks and digital measures. Fig. S4. 4AP‐related change on spatial trajectories of target reaching. [file MDS-40-2486-s001.docx]

**Supplementary Information**

**Table S1: Detailed patient characteristics**

| ID | Clinical or genetic diagnosis | Genetic specification | Sex | Age  [yr] | Onset  [yr] | Duration [yr] | FU  [mo] | PGI-C | SARA score  [BL/FU] | FARS ADL  [BL/FU] | 9HPT dom [s]  [BL/FU] | PROM-ataxia |
| --- | --- | --- | --- | --- | --- | --- | --- | --- | --- | --- | --- | --- |
| ATX02 | PNPLA6 ataxia | c.3088_3091dupAGCC / c.1635+3 G>T | F | 48 | 38 | 10 | 12 | -1 | 19/ 20.5 | 17/20 | 34/59 | 104 |
| ATX03 | SCA2 | 42 Repeats | F | 38 | 28 | 10 | 12 | -1 | 22/22 | 21/20 | 187/177 | 153 |
| ATX04 | SCA7 | 44 Repeats | M | 44 | 38 | 6 | 11 | -1 | 8/14 | 14/19 | 53/46 | - |
| ATX05 | SCA14 | c.1328T>A, VUS with positive segregation | M | 71 | 30 | 39 | 12 | 1 | 10/- | 6/5 | 41/34 | 59 |
| ATX07 | ILOCA | - | F | 54 | 49 | 5 | 12 | -1 | 16/14.5 | 16/12 | 79/73 | 127 |
| ATX08 | PIGG ataxia | c.1163G>A /  c. 2569C>T | M | 18 | 1 | 17 | 12 | 0 | 13/10.5 | 5/5 | 41/46 | - |
| ATX09 | SCA2 | 40 repeats | F | 30 | 20 | 10 | 15 | 0 | 13/10.5 | 6/11 | 38/43 | 49 |
| ATX10 | POLG ataxia (sensory + mild vermal atrophy) | c.1880G>A /  c.3521C>G | F | 29 | 22 | 7 | 13 | -1 | 4/5 | 2/2 | 20/22 | 30 |
| ATX11 | FXTAS | 64 repeats | F | 80 | 65 | 15 | 15 | -1 | 18/19 | 19/13 | 76/78 | 101 |
| ATX12 | SCA27B | 452 repeats | F | 78 | 63 | 15 | 14 | 0 | 10/8.5 | 11/14 | 29/33 | 80 |
| ATX13 | SCA19 | c.1054A>C | F | 60 | 25 | 35 | 9 | -1 | 9/7.5 | 8/- | 26/- | 58 |
| ATX16 | Episodic Ataxia Type 2 | c.4430_4431delinsA | F | 48 | 10 | 38 | 12 | -1 | 8/8.5 | 10/11 | 22/23 | 117 |
| ATX17 | SCA1 | 53 repeats | M | 31 | 24 | 7 | 12 | -1 | 18/18 | 14/14 | 72/80 | 92 |
| ATX18 | SCA15 | Macrodeletion including exon 1-12 in *ITPR1* | F | 61 | 41 | 20 | 11 | -1 | 13/12.5 | 14/16 | 42/36 | 106 |
| ATX19 | SCA15 | Macrodeletion including exon 1-12 in *ITPR1* | M | 35 | 30 | 5 | 11 | -1 | 17/15.5 | 17/16 | 109/61 | 115 |
| ATX20 | ILOCA | - | M | 57 | 49 | 8 | 11 | -1 | 13/14.5 | 11/9 | 37/34 | 104 |
| ATX21 | SCA27B | 322 repeats | M | 62 | 58 | 4 | 12 | -1 | 11/11 | 19/16 | 39/30 | 203 |
| ATX22 | SCA27B | 303 repeats | F | 71 | 60 | 11 | 12 | -1 | 6/5 | 15/13 | 26/29 | 139 |
| ATX23 | ILOCA | - | M | 61 | 51 | 10 | 14 | -1 | 11/7 | 10/10 | 34/31 | 47 |
| ATX24 | EOA (unsolved) | - | F | 53 | 38 | 15 | 12 | -1 | 7/8.5 | 8/12 | 21/25 | 101 |
| ATX25 | POLG ataxia (sensory + cerebellar with dysarthria) | c.428c>T /  c.1399G>A | F | 44 | 41 | 3 | 12 | -1 | 12/14 | 16/15 | 33/39 | 176 |
| ATX26 | Episodic Ataxia Type 2 | c.4093-3C>6 | M | 72 | 64 | 8 | 12 | 0 | 2/7.5 | 5/8 | 24/29 | 82 |
| ATX27 | SCA6 (prodromal) | blinded information | F | 57 | 57 | 0 | 11 | 0 | 3/2.5 | 1/1 | 17/17 | - |
| ATX29 | EOA with mental retardation and pyramidal signs | Two intermediate 19 CAG repeats in SCA6 | F | 56 | 32 | 24 | 12 | 0 | 12/12.5 | 23/21 | 25/31 | 139 |
| ATX30 | SCA48 | c.3G>A (de novo) | M | 36 | 30 | 6 | 12 | 0 | 18/16 | 11/10 | 61/63 | - |
| ATX31 | Mixed sensory and cerebellar ataxia with mild cognitive impairment (unsolved) | - | M | 69 | 62 | 7 | 12 | 0 | 7/7 | 5/1 | 29/28 | 14 |
| ATX32 | ILOCA | - | M | 78 | 68 | 10 | 12 | 1 | 7/6.5 | 3/5 | 27/25 | 81 |
| ATX33 | MT-ATP6 ataxia | c.314C>T | F | 70 | 57 | 13 | 14 | 1 | 10/16 | 21/20 | 27/26 | 160 |
| ATX34 | Niemann-Pick Type C | c.3019C>T /  c.3019C>T | F | 34 | 15 | 19 | 12 | 0 | 12/13.5 | 11/11 | 41/39 | 121 |
| ATX35 | SCA23 | c.414G>T | M | 72 | 65 | 7 | 12 | 0 | 7/7 | 3/3 | 28/24 | 73 |
| ATX36 | SCA5 | c.1910T>C | F | 49 | 38 | 11 | 12 | 0s | 4/3.5 | 7/5 | 22/23 | 109 |
| ATX37 | ADCA (unsolved) | - | F | 51 | 49 | 2 | 12 | -1 | 9/12.5 | 17/19 | 31/44 | 111 |
| ATX38 | ANO10 ataxia | c.132dupA /  c.1666C>T | M | 56 | 48 | 8 | 12 | -1 | 10/12 | 10/7 | 35/29 | 28 |
| ATX40 | SCA48 | c.771C>G | M | 49 | 47 | 2 | 11 | -1 | 8/9.5 | 6/10 | 31/37 | 79 |
| ATX42 | Friedreich Ataxia (sensory + cerebellar incl. dysarthria and saccadic pursuit,  mild cerebellar atrophy) | 450 / 800 repeats | F | 50 | 42 | 8 | 11 | -1 | 13/14 | -/19 | 44/42 | 114 |
| ATX43 | Friedreich Ataxia (sensory + cerebellar incl. dysarthria, saccadic pursuit, and gaze-evoked nystagmus) | 500 / 850 repeats | M | 12 | 8 | 6 | 12 | -1 | 16/- | 10/8 | 43/46 | - |

9HPT = Nine-Hole Peg-Test; ADCA = Autosomal-dominant cerebellar ataxia; ADL = Activities of daily living;
BL = Baseline; dom = dominant hand; EOA = Early-onset cerebellar ataxia; FU = Follow-up; ILOCA = Idiopathic late-onset cerebellar ataxia; mo = month; PGI-C = Patient Global Impression of Change (-1 = worsening, 0 = stable, +1 = better).

**Table S2: Correlations with PROM-Ataxia**

| **Task / Measure** | **Feature** | **Correlation to PROM-ataxia** [Spearman rho, *unadjusted p value*] | | | | | | | | | | |
| --- | --- | --- | --- | --- | --- | --- | --- | --- | --- | --- | --- | --- |
|  |  | **Total**  **Score** | **Upper Limb Composite** | **Item 8**  Rest Tremor | **Item 9**  Action Tremor | **Item 33**  Performing Tasks | **Item 34**  Control over arms | **Item 35**  Legible writing | **Item 36**  Typing on keyboard | **Item 45**  Cutting/ utensils | **Item 49**  Brushing teeth | **Item 50**  Shaving/make-up |
| **Finger Tapping** |  |  |  |  |  |  |  |  |  |  |  |  |
| *frequency* [dom] | Speed | -0.60  *<0.001* | -0.65  *<0.001* | -0.31 *0.096* | -0.48 *0.006* | -0.36  *0.046* | -0.37 *0.043* | -0.40 *0.025* | -0.30  *0.105* | -0.48  *0.006* | -0.45 *0.011* | -0.52 *0.004* |
| *frequency* [ndom] | Speed | -0.56  *0.001* | -0.60  *<0.001* | -0.31 *0.092* | -0.46 *0.009* | -0.31  *0.094* | -0.28 *0.140* | -0.31 *0.094* | -0.27  *0.143* | -0.51  *0.004* | -0.51 *0.004* | -0.54 *0.002* |
| Mean *IOI* [dom] | Speed | 0.61  *<0.001* | 0.66  *<0.001* | -0.32 *0.086* | 0.48 *0.006* | 0.35  *0.052* | 0.38 *0.039* | 0.41 *0.022* | 0.30  *0.107* | 0.48  *0.006* | 0.46 *0.009* | 0.52 *0.004* |
| Mean *IOI* [ndom] | Speed | 0.57 *0.001* | 0.61  *<0.001* | -0.32 *0.082* | 0.47 *0.008* | 0.31  *0.085* | 0.28 *0.128* | 0.31 *0.088* | 0.28  *0.132* | 0.51  *0.003* | 0.52 *0.003* | 0.55 *0.001* |
| Mean *IPI* [dom] | Speed | 0.58  *0.001* | 0.63  *<0.001* | -0.32 *0.085* | 0.41 *0.021* | 0.32  *0.080* | 0.38 *0.040* | 0.35 *0.057* | 0.34  *0.063* | 0.42  *0.018* | 0.50 *0.004* | 0.54 *0.002* |
| Mean *IPI* [ndom] | Speed | 0.48  *0.006* | 0.55  *0.001* | -0.27 *0.150* | 0.39 *0.030* | 0.30  *0.098* | 0.23 *0.220* | 0.30 *0.101* | 0.27  *0.139* | 0.47  *0.008* | 0.48 *0.007* | 0.53 *0.002* |
| Mean *ITI* [dom] | Speed | 0.46  *0.009* | 0.50  *0.004* | -0.29 *0.123* | 0.50 *0.005* | 0.46  *0.009* | 0.28 *0.137* | 0.33 *0.066* | 0.29  *0.115* | 0.41  *0.022* | 0.40 *0.027* | 0.45 *0.012* |
| Mean *ITI* [ndom] | Speed | 0.49  *0.005* | 0.50  *0.004* | -0.29 *0.118* | 0.50 *0.004* | 0.41  *0.021* | 0.24 *0.206* | 0.27 *0.139* | 0.27  *0.142* | 0.49  *0.006* | 0.45 *0.012* | 0.49 *0.006* |
| Mean *TD* [dom] | Speed | 0.55  *0.001* | 0.61  *<0.001* | -0.37 *0.044* | 0.43 *0.015* | 0.18  *0.332* | 0.31 *0.095* | 0.34 *0.063* | 0.21  *0.251* | 0.42  *0.019* | 0.42 *0.019* | 0.49 *0.006* |
| Mean *TD* [ndom] | Speed | 0.59  *0.001* | 0.63  *<0.001* | 0.38 *0.038* | 0.42 *0.018* | 0.13  *0.492* | 0.24 *0.195* | 0.29 *0.117* | 0.20  *0.285* | 0.49  *0.005* | 0.57 *0.001* | 0.58 *0.001* |
| Mean *T_fall_* [dom] | Speed | 0.58  *0.001* | 0.61  *<0.001* | 0.35 *0.059* | 0.37 *0.042* | 0.17  *0.365* | 0.31 *0.090* | 0.29 *0.109* | 0.22  *0.235* | 0.42  *0.019* | 0.52 *0.003* | 0.59 *0.001* |
| Mean *T_fall_* [ndom] | Speed | 0.55  *0.001* | 0.58  *0.001* | 0.33 *0.079* | 0.30 *0.095* | 0.08  *0.671* | 0.23 *0.217* | 0.28 *0.129* | 0.20  *0.290* | 0.50  *0.004* | 0.54 *0.002* | 0.57 *0.001* |
| STD *T_rise_* [ndom] | Variability | 0.59  *<0.001* | 0.70  *<0.001* | 0.42 *0.020* | 0.42 *0.017* | 0.44  *0.012* | 0.53 *0.003* | 0.41 *0.022* | 0.56  *0.001* | 0.57  *0.001* | 0.60  < *0.001* | 0.58 *0.001* |
| **Diadochokinesia** |  |  |  |  |  |  |  |  |  |  |  |  |
| *frequency* [ndom] | Speed | -0.45  *0.011* | -0.42  *0.020* | -0.14 *0.449* | -0.21 *0.246* | 0.01  *0.953* | -0.37 *0.046* | -0.12 *0.538* | -0.10  *0.594* | -0.31  *0.090* | -0.30 *0.095* | -0.39 *0.033* |
| **Diadochokinesia (cont.)** |  | **Total**  **Score** | **Upper Limb Composite** | **Item 8**  Rest Tremor | **Item 9**  Action Tremor | **Item 33**  Performing Tasks | **Item 34**  Control over arms | **Item 35**  Legible writing | **Item 36**  Typing on keyboard | **Item 45**  Cutting/ utensils | **Item 49**  Brushing teeth | **Item 50**  Shaving/make-up |
| Mean *IOI* [ndom] | Speed | 0.46  *0.010* | 0.42  *0.017* | 0.15 *0.433* | 0.22 *0.228* | -0.01  *0.967* | 0.38 *0.038* | 0.12 *0.538* | 0.11  *0.572* | 0.32  *0.081* | 0.31 *0.084* | 0.40 *0.028* |
| Mean *IPI* [ndom] | Speed | 0.41  *0.021* | 0.36  *0.044* | 0.16 *0.399* | 0.20 *0.289* | -0.03  *0.863* | 0.38 *0.038* | 0.08 *0.669* | 0.07  *0.723* | 0.29  *0.112* | 0.27 *0.146* | 0.35 *0.058* |
| STD *IOI* [ndom] | Variability | 0.22  *0.241* | 0.40  *0.024* | 0.16 *0.399* | 0.34 *0.063* | 0.37  *0.041* | 0.37 *0.044* | 0.31 *0.086* | 0.34  *0.065* | 0.19  *0.298* | 0.09 *0.632* | 0.24 *0.196* |
| STD *IPI* [ndom] | Variability | 0.31  *0.092* | 0.48  *0.007* | 0.17 *0.370* | 0.35 *0.054* | 0.39  *0.030* | 0.43 *0.019* | 0.36 *0.044* | 0.36  *0.049* | 0.26  *0.155* | 0.13 *0.482* | 0.29 *0.122* |
| STD *ITI* [ndom] | Variability | 0.20  *0.269* | 0.38  *0.037* | 0.23 *0.227* | 0.32 *0.078* | 0.37  *0.043* | 0.38 *0.036* | 0.33 *0.070* | 0.35  *0.057* | 0.17  *0.361* | 0.12 *0.514* | 0.25 *0.180* |
| STD *T_fall_* [ndom] | Variability | 0.54  *0.002* | 0.62  *<0.001* | 0.24 *0.193* | 0.43 *0.016* | 0.17  *0.360* | 0.51 *0.004* | 0.32 *0.081* | 0.34  *0.062* | 0.33  *0.066* | 0.36 *0.044* | 0.37 *0.042* |
| **Grip-Lift** |  |  |  |  |  |  |  |  |  |  |  |  |
| *orientation index* [ndom] | Stability | 0.39  *0.030* | 0.52  *0.003* | 0.65  < *0.001* | 0.58 *0.001* | 0.32  *0.080* | 0.26 *0.159* | 0.38 *0.037* | 0.19  *0.309* | 0.40  *0.025* | 0.41 *0.021* | 0.56 *0.001* |
| *position index* [dom] | Stability | 0.34  *0.065* | 0.46  *0.008* | 0.49 *0.005* | 0.49 *0.005* | 0.26  *0.156* | 0.17 *0.375* | 0.39 *0.028* | 0.26  *0.163* | 0.42  *0.020* | 0.27 *0.149* | 0.46 *0.011* |
| *position index* [ndom] | Stability | 0.47  *0.008* | 0.49  *0.005* | 0.60 *0.001* | 0.59 *0.001* | 0.24  *0.194* | 0.25 *0.181* | 0.23 *0.220* | 0.15  *0.422* | 0.38  *0.034* | 0.44 *0.014* | 0.59 *0.001* |
| **Spiral Drawing** |  |  |  |  |  |  |  |  |  |  |  |  |
| *SPARC* | Smoothness | -0.26  *0.160* | -0.46  *0.009* | -0.43 *0.018* | -0.39 *0.028* | -0.21  *0.264* | -0.33 *0.077* | -0.50 *0.004* | -0.23  *0.210* | -0.32  *0.081* | -0.24 *0.195* | -0.35 *0.058* |
| *acc_prc90-100_* | Smoothness | 0.17  *0.350* | 0.42  *0.019* | -0.24 *0.207* | 0.43 *0.017* | 0.30  *0.105* | 0.38 *0.040* | 0.51 *0.003* | 0.20  *0.282* | 0.25  *0.166* | 0.08 *0.669* | 0.21 *0.270* |
| *speed_prc90-100_* | Smoothness | 0.29  *0.114* | 0.45  *0.011* | -0.24 *0.208* | 0.32 *0.075* | 0.12  *0.508* | 0.35 *0.055* | 0.45 *0.012* | 0.16  *0.396* | 0.29  *0.111* | 0.22 *0.232* | 0.33 *0.072* |
| **Target Reaching** |  |  |  |  |  |  |  |  |  |  |  |  |
| *path_3D_* | Efficiency | 0.41  *0.023* | 0.46  *0.010* | 0.22 *0.252* | 0.41 *0.022* | 0.08  *0.683* | 0.31 *0.097* | 0.28 *0.132* | 0.18  *0.339* | 0.42  *0.019* | 0.32 *0.079* | 0.39 *0.034* |
| *path_2D_* | Efficiency | 0.32  *0.077* | 0.41  *0.024* | 0.26 *0.161* | 0.45 *0.012* | 0.07  *0.709* | 0.22 *0.232* | 0.29 *0.114* | 0.14  *0.457* | 0.36  *0.046* | 0.27 *0.135* | 0.35 *0.055* |
| *path_lr_* | Efficiency | 0.38  *0.035* | 0.45  *0.010* | 0.17 *0.368* | 0.38 *0.033* | 0.06  *0.732* | 0.35 *0.057* | 0.33 *0.074* | 0.22  *0.237* | 0.35  *0.057* | 0.25 *0.168* | 0.34 *0.067* |
| **Target Reaching (cont.)** |  | **Total**  **Score** | **Upper Limb Composite** | **Item 8**  Rest Tremor | **Item 9**  Action Tremor | **Item 33**  Performing Tasks | **Item 34**  Control over arms | **Item 35**  Legible writing | **Item 36**  Typing on keyboard | **Item 45**  Cutting/ utensils | **Item 49**  Brushing teeth | **Item 50**  Shaving/make-up |
| *dysmetria* | Endpoint precision | 0.36  *0.044* | 0.44  *0.012* | 0.33 *0.074* | 0.52 *0.003* | 0.12  *0.506* | 0.23 *0.220* | 0.31 *0.094* | 0.13  *0.482* | 0.41  *0.021* | 0.29 *0.110* | 0.38 *0.037* |
| *SPARC_3D_* | Smoothness | -0.36  *0.047* | -0.39  *0.031* | -0.41 *0.025* | -0.44 *0.013* | -0.01  *0.965* | -0.21 *0.263* | -0.27 *0.149* | -0.10  *0.585* | -0.31  *0.088* | -0.27 *0.135* | -0.38 *0.036* |
| *latency_3D,acc_* | Speed | 0.28  *0.129* | 0.36  *0.048* | 0.24 *0.206* | 0.45 *0.011* | 0.34  *0.064* | 0.16 *0.411* | 0.31 *0.092* | 0.16  *0.375* | 0.27  *0.140* | 0.11 *0.552* | 0.29 *0.122* |
| *latency_2D,acc_* | Speed | 0.43  *0.016* | 0.52  *0.003* | 0.34 *0.068* | 0.49 *0.005* | 0.26  *0.161* | 0.29 *0.126* | 0.36 *0.044* | 0.24  *0.202* | 0.34  *0.063* | 0.31 *0.086* | 0.44 *0.015* |
| *latency_3D,acc,MAD_* | Variability | 0.29  *0.115* | 0.39  *0.032* | 0.30 *0.104* | 0.42 *0.018* | 0.27  *0.147* | 0.27 *0.156* | 0.38 *0.033* | 0.28  *0.125* | 0.19  *0.295* | 0.12 *0.505* | 0.28 *0.136* |
| *latency_2D,acc,MAD_* | Variability | 0.33  *0.068* | 0.40  *0.027* | 0.37 *0.044* | 0.37 *0.042* | 0.12  *0.518* | 0.23 *0.215* | 0.36 *0.046* | 0.30  *0.096* | 0.27  *0.144* | 0.24 *0.196* | 0.36 *0.050* |
| *latency_2D,dec,MAD_* | Variability | 0.38  *0.034* | 0.40  *0.026* | 0.28 *0.137* | 0.39 *0.029* | 0.07  *0.707* | 0.38 *0.039* | 0.26 *0.163* | 0.23  *0.217* | 0.26  *0.160* | 0.20 *0.274* | 0.28 *0.127* |
| *ITI_MAD_* | Variability | 0.40  *0.025* | 0.38  *0.037* | -0.21 *0.264* | 0.34 *0.063* | -0.06  *0.766* | 0.31 *0.091* | 0.24 *0.187* | 0.22  *0.233* | 0.22  *0.237* | 0.15 *0.429* | 0.19 *0.304* |
| *path_3D,MAD_* | Variability | 0.32  *0.075* | 0.38  *0.036* | -0.31 *0.090* | 0.39 *0.029* | -0.02  *0.934* | 0.28 *0.134* | 0.26 *0.156* | 0.20  *0.279* | 0.30  *0.101* | 0.25 *0.169* | 0.29 *0.114* |
| *path_2D,MAD_* | Variability | 0.31  *0.091* | 0.39  *0.029* | 0.33 *0.072* | 0.37 *0.038* | 0.03  *0.861* | 0.21 *0.277* | 0.33 *0.071* | 0.23  *0.204* | 0.30  *0.096* | 0.25 *0.177* | 0.33 *0.073* |
| *path_v,MAD_* | Variability | 0.35  *0.050* | 0.42  *0.020* | 0.39 *0.032* | 0.40 *0.025* | 0.02  *0.906* | 0.26 *0.171* | 0.28 *0.125* | 0.14  *0.451* | 0.30  *0.099* | 0.29 *0.109* | 0.38 *0.040* |
| *dtw_3D_* | Variability | 0.37  *0.038* | 0.40  *0.028* | 0.42 *0.020* | 0.49 *0.005* | 0.08  *0.657* | 0.28 *0.135* | 0.32 *0.081* | 0.25  *0.181* | 0.28  *0.128* | 0.26 *0.163* | 0.33 *0.071* |
| *dtw_2D_* | Variability | 0.35  *0.054* | 0.39  *0.028* | 0.41 *0.025* | 0.50 *0.004* | 0.12  *0.528* | 0.27 *0.153* | 0.35 *0.056* | 0.24  *0.189* | 0.28  *0.131* | 0.24 *0.199* | 0.32 *0.082* |
| **Clinical Outcome** |  |  |  |  |  |  |  |  |  |  |  |  |
| 9HPT [dom] | - | 0.14  *0.468* | 0.27  *0.145* | 0.19 *0.327* | 0.37 *0.039* | 0.15  *0.426* | 0.20 *0.289* | 0.17 *0.361* | 0.16  *0.380* | 0.36  *0.048* | 0.19 *0.313* | 0.22 *0.235* |
| 9HPT [ndom] | - | 0.22  *0.241* | 0.38  *0.041* | 0.28 *0.137* | 0.39 *0.029* | 0.07  *0.707* | 0.38 *0.039* | 0.26 *0.163* | 0.23  *0.217* | 0.26  *0.160* | 0.20 *0.274* | 0.28 *0.127* |

3D/2D = 3D trajectory and virtual projection on 2D plane; 9HPT = 9-hole peg-test; dom/ndom = dominant/non-dominant hand; acc = acceleration; dec = deceleration;
dtw = dynamic time warp; IOI = inter-onset interval; IPI = inter-peak interval; ITI = inter-tap interval; MAD = median absolute deviation; MED = median; STD = standard deviation; SPARC = Spectral Arc Length; TD = tap duration; *T_rise_* = duration of tap until peak force; *T_fall_* = duration of tap after peak force.

**Table S3: Test-retest reliability across digital motor tasks and measures**

| **Measure** | **Unit** | **Feature** | **ICC** | **SRD** (% range) | **Learning effect** (% range) | **SEM** | **MDC_90_** | **MDC_95_** | **MDC_95_** (group) |
| --- | --- | --- | --- | --- | --- | --- | --- | --- | --- |
| **Finger Tapping** |  |  |  |  |  |  |  |  |  |
| *frequency* [dom] | Hz | Speed | 0.98 | 8.40 | 0.21 | 0.10 | 0.24 | 0.28 | 0.075 |
| *frequency* [ndom] | Hz | Speed | 0.99 | 8.32 | -0.99 | 0.16 | 0.37 | 0.44 | 0.119 |
| Mean *IOI* [dom] | s | Speed | 0.99 | 6.48 | -0.39 | 0.01 | 0.02 | 0.02 | 0.006 |
| Mean *IOI* [ndom] | s | Speed | 0.99 | 7.11 | 0.69 | 0.01 | 0.03 | 0.03 | 0.008 |
| Mean *IPI* [dom] | s | Speed | 0.98 | 8.18 | 0.08 | 0.01 | 0.02 | 0.02 | 0.005 |
| Mean *IPI* [ndom] | s | Speed | 0.98 | 8.85 | 0.81 | 0.01 | 0.02 | 0.03 | 0.007 |
| Mean *ITI* [dom] | 4s | Speed | 0.96 | 10.72 | 2.84 | 0.01 | 0.09 | 0.02 | 0.004 |
| Mean *ITI* [ndom] | s | Speed | 0.97 | 10.51 | 2.67 | 0.01 | 0.02 | 0.02 | 0.005 |
| Mean *TD* [ndom] | s | Speed | 0.97 | 12.12 | -1.40 | 0.01 | 0.02 | 0.03 | 0.007 |
| Mean *T_fall_* [ndom] | s | Speed | 0.96 | 11.50 | -0.88 | 0.01 | 0.01 | 0.01 | 0.003 |
| **Diadochokinesia** |  |  |  |  |  |  |  |  |  |
| *frequency* [ndom] | Hz | Speed | 0.98 | 10.08 | 2.66 | 0.12 | 0.27 | 0.32 | 0.089 |
| Mean *IOI* [ndom] | s | Speed | 0.99 | 7.67 | -2.02 | 0.03 | 0.06 | 0.07 | 0.020 |
| Mean *IPI* [ndom] | s | Speed | 0.99 | 8.47 | -1.94 | 0.02 | 0.05 | 0.06 | 0.018 |
| **Grip-Lift** |  |  |  |  |  |  |  |  |  |
| *position index* [dom] | log_10_ [cm/s] | Stability | 0.96 | 9.82 | -0.72 | 0.09 | 0.21 | 0.25 | 0.068 |
| **Spiral Drawing** |  |  |  |  |  |  |  |  |  |
| *acc_prc90-100_* | log_10_ [cm/s^2^] | Smoothness | 0.96 | 13.62 | 2.74 | 0.09 | 0.21 | 0.25 | 0.070 |
| **Target Reaching** |  |  |  |  |  |  |  |  |  |
| *path_3D_* | cm | Efficiency | 0.95 | 9.77 | -1.71 | 1.18 | 2.76 | 3.27 | 0.875 |
| *path_2D_* | cm | Efficiency | 0.91 | 12.02 | -1.93 | 0.80 | 1.86 | 2.21 | 0.592 |
| *path_lr_* | cm | Efficiency | 0.93 | 11.01 | -1.20 | 0.58 | 1.35 | 1.60 | 0.429 |
| *SPARC_3D_* | log_10_ \|[ ]\| | Smoothness | 0.97 | 8.00 | 0.23 | 0.01 | 0.02 | 0.03 | 0.007 |
| *latency_2D,acc_* | ms | Speed | 0.96 | 11.84 | -0.35 | 22.18 | 51.75 | 61.48 | 16.43 |
| *latency_2D,acc,MAD_* | log_10_ [ms] | Variability | 0.96 | 13.03 | -0.58 | 0.13 | 0.30 | 0.36 | 0.095 |
| *path_2D,MAD_* | log_10_ [mm] | Variability | 0.94 | 13.80 | -0.66 | 0.15 | 0.36 | 0.42 | 0.113 |
| *dtw_3D_* | log_10_ [cm/s] | Variability | 0.96 | 10.41 | -1.69 | 0.08 | 0.19 | 0.22 | 0.059 |
| *dtw_2D_* | log_10_ [cm/s] | Variability | 0.96 | 10.81 | -2.06 | 0.08 | 0.19 | 0.23 | 0.061 |

3D/2D = 3D movement trajectory and virtual projection on 2D plane; dom/ndom = dominant/non-dominant hand;
acc = acceleration; dec = deceleration; IOI = inter-onset interval; IPI = inter-peak interval; ITI = inter-tap interval; MAD = median absolute deviation; MDC_90/95_ = minimal detectable change for within-patient change, based on retest distribution of ataxia patients; MDC_90_ (group) = minimal detectable change for between-group difference; MED = median; STD = standard deviation; SPARC = Spectral Arc Length; SRD = smallest real difference; TD = tap duration; T_fall_ = duration of the tap after reaching peak force.

**Table S4: Sensitivity to longitudinal change across all digital measures validated for significant correlations with PROM-ataxia and for test-retest reliability**

| **Task**  Feature  *Measure* | **Feature** | **Unit** | **Paired difference at 1-year follow-up** | | | | | | |
| --- | --- | --- | --- | --- | --- | --- | --- | --- | --- |
|  |  |  | **Healthy Controls** | **Ataxia patients by PGI-C** | | | | | |
|  |  |  |  | *all* | | *progression* | | *no progression* | |
|  |  |  | r_prb_ | r_prb_ /SRM | m(std) | r_prb_  /SRM | MIC*_AC_* | r_prb_ | MIC*_95_* |
| **Finger Tapping** |  |  |  |  |  |  |  |  |  |
| *frequency* [dom] | Speed | s | n.s. | n.s. | -0.14  (0.36) | n.s. | - | n.s. | -0.41 |
| *frequency* [ndom] | Speed | s | n.s. | -0.45*  /-0.34^#^ | -0.13  (0.39) | -0.49*  /-0.39^#^ | -0.16 | n.s. | -0.26 |
| Mean *IOI* [dom] | Speed | s | n.s. | n.s. | 0.01 (0.04) | n.s. | - | n.s. | 0.02 |
| Mean *IOI* [ndom] | Speed | s | n.s. | 0.38*  /0.14 | 0.01  (0.05) | n.s. | - | n.s. | 0.02 |
| Mean *IPI* [dom] | Speed | s | n.s. | n.s. | 0.01 (0.04) | n.s. | - | n.s. | 0.02 |
| Mean *IPI* [ndom] | Speed | s | n.s. | n.s. | 0.01 (0.05) | 0.42*  /0.12 | 0.01 | n.s. | 0.02 |
| Mean *ITI* [dom] | Speed | s | n.s. | n.s. | 0.002 (0.03) | n.s. | - | n.s. | 0.01 |
| Mean *ITI* [ndom] | Speed | s | n.s. | n.s. | -0.001 (0.04) | n.s. | - | n.s. | 0.01 |
| Mean *TD* [ndom] | Speed | s | n.s. | 0.40*  /0.31 | 0.01  (0.03) | n.s. | - | n.s. | 0.02 |
| Mean *T_fall_* [ndom] | Speed | s | n.s. | n.s. | 0.003 (0.01) | n.s. | - | n.s. | 0.01 |
| **Diadochokinesia** |  |  |  |  |  |  |  |  |  |
| *frequency* [ndom] | Speed | Hz | n.s. | -0.38*  /-0.37^#^ | -0.09  (0.24) | -0.44*  /-0.41^#^ | -0.08 | n.s. | -0.24 |
| Mean *IOI* [ndom] | Speed | s |  |  |  |  |  |  |  |
| Mean *IPI* [ndom] | Speed | s | n.s. | 0.42*  /0.31 | 0.03  (0.08) | 0.42* /0.28 | 0.03 | n.s. | 0.05 |
| **Grip-Lift** |  |  |  |  |  |  |  |  |  |
| *position index* [dom] | Stability | log10 [mm/s] | n.s. | n.s. | -0.04 (0.39) | n.s. | - | n.s. | 0.23 |
| **Spiral Drawing** |  |  |  |  |  |  |  |  |  |
| *acc_prc-90-100_* | Smoothness | log_10_ [mm/s^2^] | n.s. | n.s. | 0.03  (0.27) | 0.47* /0.37 | 0.08 | n.s. | 0.09 |
| **Target Reaching** |  |  |  |  |  |  |  |  |  |
| *path_3D_* | Efficiency | cm | n.s. | n.s. | 0.43 (1.77) | n.s. | - | n.s. | 1.03 |
| *path_2D_* | Efficiency | cm | n.s. | n.s. | 0.27 (0.89) | n.s. | - | n.s. | 0.27 |

| **Task**  Feature  *Measure* | **Feature** | **Unit** | **Paired difference at 1-year follow-up** | | | | | | |
| --- | --- | --- | --- | --- | --- | --- | --- | --- | --- |
|  |  |  | **Healthy Controls** | **Ataxia patients by PGI-C** | | | | | |
|  |  |  |  | *all* | | *progression* | | *no progression* | |
|  |  |  | r_prb_  /SRM | r_prb_ /SRM | m(std) | r_prb_ /SRM | MIC*_AC_* | r_prb_ | MIC*_95_* |
| **Target Reaching**  **(continued)** |  |  |  |  |  |  |  |  |  |
| *path_lr_* | Efficiency | cm | n.s. | n.s. | 0.09 (0.52) | n.s. | - | n.s. | 0.33 |
| *SPARC_3D_* | Smoothness | log_10_  \|[]\| | n.s. | 0.51*  /0.52^#^ | 0.01  (0.02) | n.s. | - | n.s. | 0.02 |
| *latency_2D,acc_* | Speed | ms | -0.75*  /-0.82^#^ | n.s. | -3.92 (31.9) | n.s. | - | - | 20.8 |
| *latency_2D,acc,MAD_* | Variability | log_10_ [ms] | n.s. | n.s. | 0.02 (0.39) | n.s. | - | n.s. | 0.24 |
| *path_2D,MAD_* | Variability | log_10_ [mm] | n.s. | n.s. | -0.10 (0.32) | n.s. | - | n.s. | 0.12 |
| *dtw_2D_* | Variability | log_10_ [cm/s] | n.s. | n.s. | -0.04 (0.26) | n.s. | - | n.s. | 0.05 |
| *dtw_3D_* | Variability | log_10_ [cm/s] | n.s. | n.s. | -0.01 (0.26) | n.s. | - | n.s. | 0.06 |
| **Clinical Outcomes** |  |  |  |  |  |  |  |  |  |
| SARA | - | points | - | n.s. | 0.75  (2.28) | 0.54*  /0.44 | 0.91 | n.s. | 2.32 |
| FARS ADL | - | points | - | 0.49* /0.38^#^ | 0.96  (2.51) | 0.48* /0.39^#^ | 1.05 | n.s. | 2.14 |
| 9HPT [dom] | - | 1/s | 0.65* /0.65 | n.s. | -0.001  (0.007) | n.s. | - | n.s. | 0.002 |
| 9HPT [ndom] | - | 1/s | - | n.s. | 0.001  (0.005) | n.s. | - | n.s. | 0.004 |
|  |  |  |  |  |  |  |  |  |  |

9HPT = 9-hole peg-test; acc = acceleration; dom/ndom = dominant/non-dominant hand; FARS-ADL = Friedreich Ataxia Rating Scale Activities of Daily Living; IOI = inter-onset interval; IPI = inter-peak interval; ITI = inter-tap interval; m= mean; MIC_AC_ = minimal important change, based on average change; MIC_95_ = minimal important change, based on 95% confidence interval; n.s. = not significant; PGI-C = Patient Global Impression of Change; SARA= Scale for the Assessment and Rating of Ataxia; std = standard deviation; SPARC = Spectral Arc Length; SRM = standardized response mean (mean/std of change), with measures passing prior Shapiro-Wilk test for normality distribution of change highlighted by^#^; TD = tap duration. *p<0.05 in Wilcoxon signed rank test.

**Figure S1: Bland-Altmann plots of test-retest assessment**

**
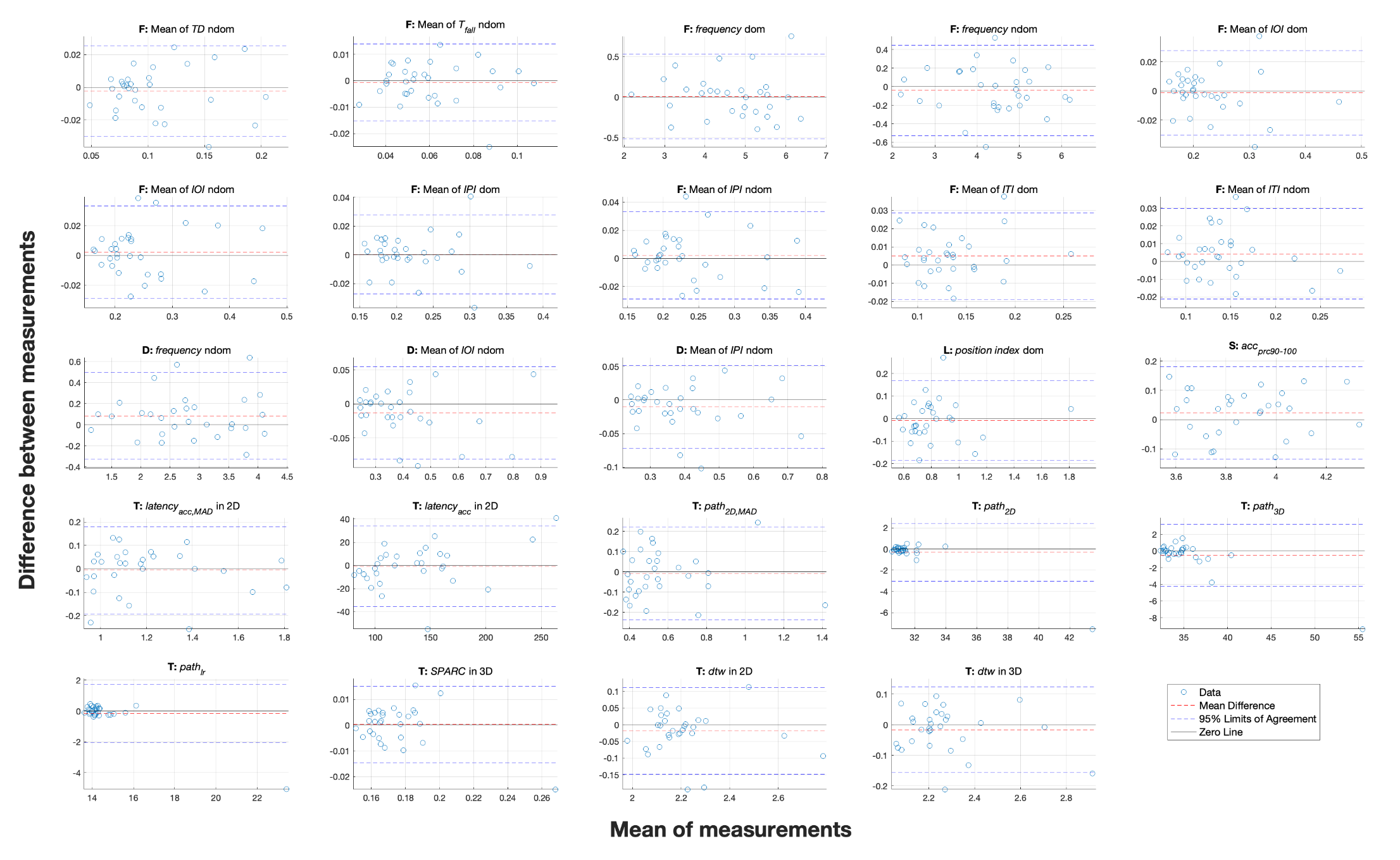
**

**Figure S1: Bland-Altmann plots of test-retest assessment**. Plot of difference against mean of digital measure between test and 2-week retest assessment in 31 subjects (14 ataxia patients, 17 healthy controls). Dashed lines indicate mean (red) and two standard deviations (blue) across the cohort. Illustrated digital measures passed restrictive validation for test-retest reliability, with excellent intraclass correlation coefficient (ICC: 0.91-0.99), smallest real differences (SRD: <14% relative to a measure’s range), and learning effects (<3% change relative to a measure’s range) [Kanzler, C. M., et al. (2020). "A data-driven framework for selecting and validating digital health metrics: use-case in neurological sensorimotor impairments." NPJ digital medicine 3(1): 1-17.]

**Figure S2: Distribution of longitudinal change**


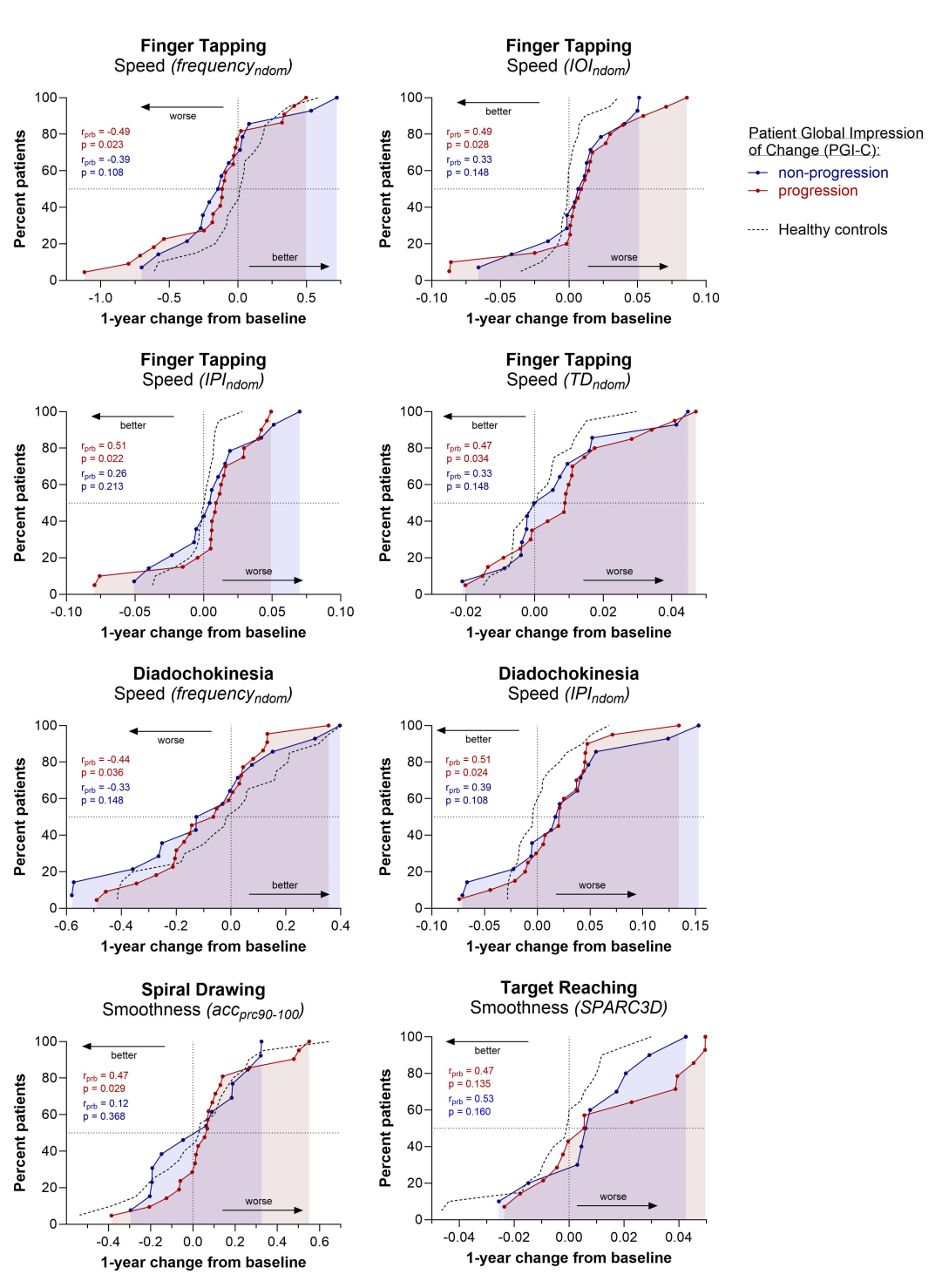


**Figure S2: Distribution of longitudinal change**. Cumulative distribution of change (1-year follow-up – baseline) across ataxia patients, stratified by subjective worsening (red) vs. stability/improvement (blue) according to Patient Global Impression of Change. Grubb’s method was applied to identify and remove outliers in finger tapping (ATX02, ATX21), diadochokinesia (ATX03, ATX21), and spiral drawing (ATX30). Illustrated measures showed significant worsening between baseline and follow-up across all patients and/or specifically in patients with worsening PGI-C (Wilcoxon signed rank-test, r_prb_ = matched-pairs rank biserial correlations). Note the consistent shift between progressors and healthy controls (dashed lines) crossing the zero-change intersect, while changes in non-progressors range from overlaps with healthy controls (indicating not change) to overlaps with progressors (indicating that a measure captures change not perceived by the patient). *accprc_90-10_* = highest decile of instantaneous accelerations; *ndom* = non-dominant hand; *IOI* = inter-onset interval; *IPI* = inter-peak interval; *SPARC3D* = spectral arc length of 3D reaching trajectory; *TD* = tap duration.

**Figure S3: 4AP-related change in SCA27B across motor tasks and digital measures**


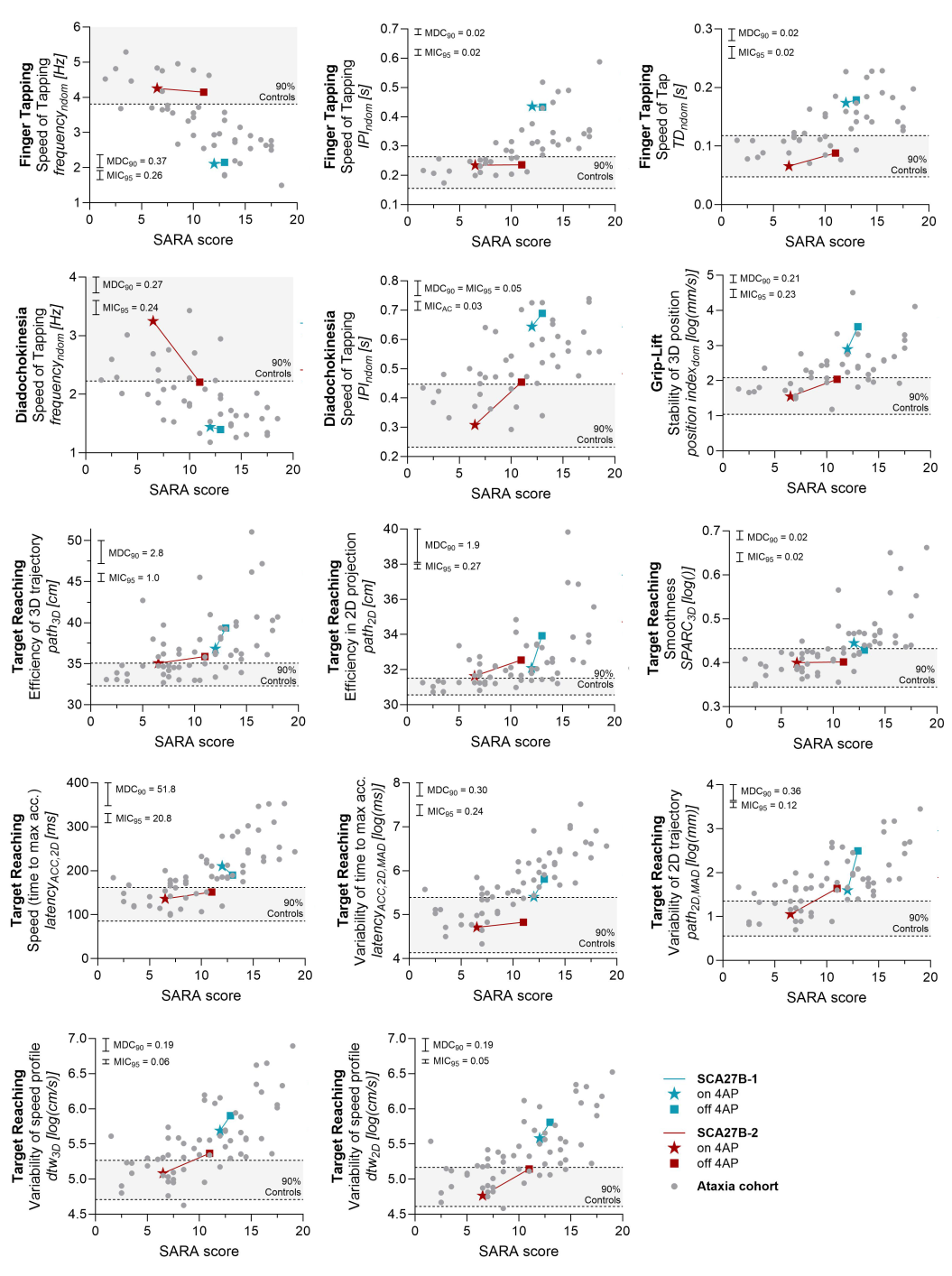


**Figure S3: 4AP-related change in SCA27B across motor tasks and digital measures**. Scatter plot of digital measures against ataxia severity in the SARA, with intra-individual comparison of two SCA27B patients on vs. off treatment (color) relative to correlations among the cross-genotype ataxia cohort and the 90% percentile of the distribution of healthy controls (shaded area). Unless changes after withdrawal of 4AP remain below MDC and MIC thresholds (error bars for visual comparison), all digital measures across several tasks consistently indicate worsening of upper limb ataxia after withdrawal of 4AP. ACC = acceleration; *dtw* = dynamic time warp; MAD = median absolute deviation; MDC_90_ = minimal detectable change; MIC_95_ = minimal important change, based on 95% confidence interval in patients with subjective stability over 1 year; SARA = Scale for the Assessment and Rating of Ataxia; *IPI* = inter-peak interval; *SPARC* = spectral arc length; *TD* = tap duration.

**Figure S4: 4AP-related change on spatial trajectories of target reaching**


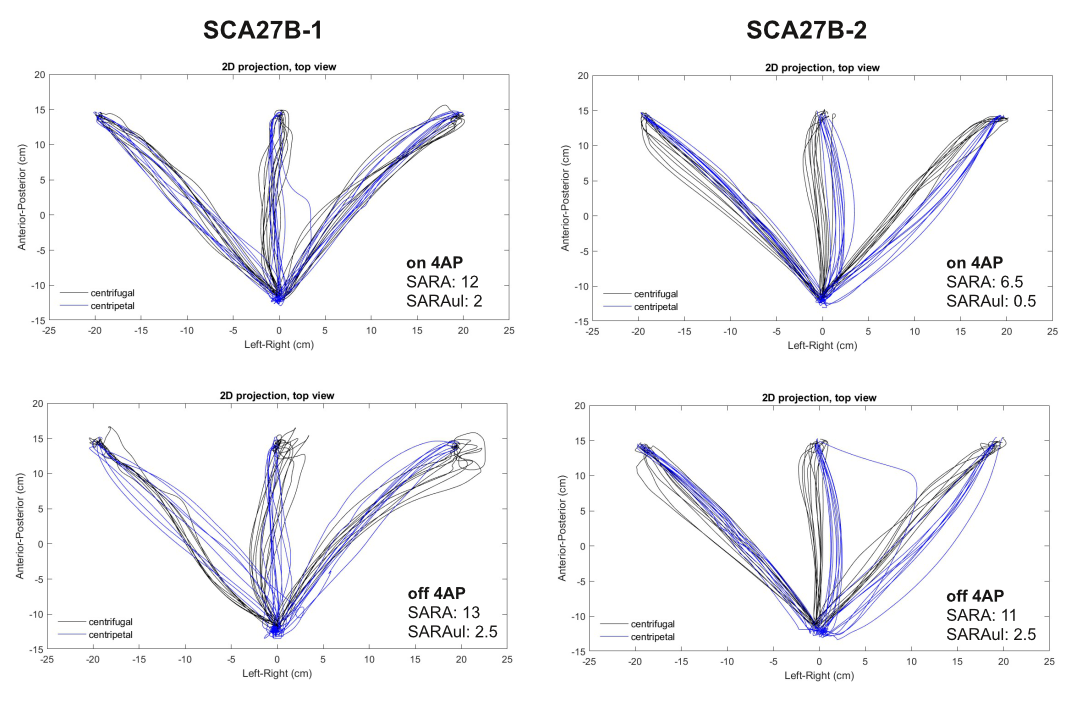


**Figure S4: 4AP-related change on spatial trajectories of target reaching**. 2D projection of 10 alternating reaching movements between four targets in centrifugal (grey) and centripetal (blue) direction, which are used to calculate the spatial variability of target reaching by means of the median absolute deviation of the 2D path length *(path_2D,MAD_)*. The four trajectories illustrate target reaching on treatment (top row) vs. off treatment (bottom row) with 4-aminopyridine (4AP) in two patients with SCA27B. After withdrawal of 4AP, spatial variability qualitatively increases in SCA27B-1 despite minor changes in SARA or its upper limb composite (SARAul), while no evident qualitative change can be seen in SCA27B-2 despite larger changes in SARA and SARAul. Nevertheless, quantitative motor assessment by (the logarithm of) *path_2D,MAD_* could capture worsening of spatial variability above minimal detectable change and minimal important change thresholds in both patients [see Fig. 4 and S3].
